# Contaminated drinking water facilitates *Escherichia coli* strain-sharing within households in urban informal settlements

**DOI:** 10.1038/s41564-025-01986-w

**Published:** 2025-05-01

**Authors:** Daehyun D. Kim, Jenna M. Swarthout, Colin J. Worby, Benard Chieng, John Mboya, Ashlee M. Earl, Sammy M. Njenga, Amy J. Pickering

**Affiliations:** 1 Department of Civil and Environmental Engineering, University of California, Berkeley, CA USA; 2 Department of Civil and Environmental Engineering, Tufts University, Medford, MA USA; 3 Infectious Disease and Microbiome Program, Broad Institute, Cambridge, MA USA; 4 Kenya Medical Research Institute, Nairobi, Kenya; 5 Chan Zuckerberg Biohub—San Francisco, San Francisco, CA USA; 6 Blum Center for Developing Economies, University of California, Berkeley, Berkeley, CA USA

**Keywords:** Bacterial infection, Metagenomics, Water microbiology

## Abstract

Identifying bacterial transmission pathways is crucial to inform strategies that limit the spread of pathogenic and antibiotic-resistant bacteria. Here we assessed *Escherichia coli* strain-sharing and overlap of antibiotic resistance genes (ARGs) across humans, poultry, canines, soil, and drinking water within and between households in urban informal settlements in Nairobi, Kenya. We collected 321 samples from 50 households with half having access to chlorinated water. We performed Pooling Isolated Colonies-seq, which sequences pools of up to five *E. coli* colonies per sample to capture strain diversity. Pooling Isolated Colonies-seq captured 1,516 colonies and identified 154 strain-sharing events, overcoming limitations of single-isolate sequencing and metagenomics. Within households, strain-sharing rates and resistome similarities across sample types were strongly correlated, suggesting clonal transmission of ARGs. *E. coli* isolated from the environment carried clinically relevant ARGs. Strain-sharing was rare between animals and humans but frequent between humans and drinking water. *E. coli*-contaminated stored drinking water was associated with higher human–human strain-sharing within households. These results suggest that contaminated drinking water facilitates human to human strain-sharing, and water treatment can disrupt transmission.

## Abstract (web summary)

*Escherichia coli*-contaminated stored drinking water facilitates strain-sharing between household members, suggesting water treatment can curb bacterial transmission and antibiotic resistance in urban low-income communities.

## Main

Bacterial enteric infections cause 9.2% of annual deaths worldwide in children under the age of 5 years^1,2^. Bacterial infections can trigger acute gastrointestinal illness, while asymptomatic infections are associated with child growth faltering^3,4^. The incidence and disease burden of bacterial infections are highest in rapidly urbanizing low- and middle-income countries^5^, where over 50% of the urban population live in informal urban settings with limited access to safe drinking water, sanitation and appropriate hygiene (WASH) infrastructure^6^. Furthermore, in these informal urban settings, families often engage in small-scale animal husbandry within confined spaces, increasing the risk of zoonotic transmission^7,8^. The high prevalence of infectious diseases in urban informal settlements, coupled with unregulated antibiotic use, promotes both the emergence of new antibiotic resistance gene (ARGs) variants through the proliferation of antibiotic-resistant bacteria and the spread of antibiotic resistance between bacterial strains via horizontal gene transfer^6,9^. A recent meta-analysis of faecal metagenomes from 26 countries^10^ identified a higher abundance of ARGs in urban areas in Africa and South East Asia compared to rural areas.

Enteric pathogens are transmitted to humans by direct contact with infected hosts or via exposure to faecally contaminated environments^11^. Exposure to animals has recently garnered attention as a risk factor for enteric infections in low- and middle-income countries with reports of strain-sharing between humans and domestic or wild animals that are in close proximity^12,13^. The previous studies have mainly focused on examining bacterial strain-sharing between humans and animals^14,15^, without investigating the environmental exposure pathways through which transmission occurs^16^. However, humans are regularly exposed to environmental microbiota through consuming drinking water, hand-to-mouth contact with soil and inhaling soil dust^7,14^. Understanding transmission pathways across humans, animals and the environment, along with their spatial range, is important to determine how bacterial transmission could be interrupted and the scale at which interventions need to be implemented^17^.

The epidemiological study of bacterial transmission has traditionally been conducted by sequencing whole genomes of individual isolates selectively cultured from each host^18^. Sequencing individual isolates has the advantage that it can facilitate the comparison of complete genomes at high resolution, using metrics such as core genome multilocus sequence typing^19^ or core genome single nucleotide polymorphisms^20^. However, due to the labour and cost burden of culturing and sequencing isolates, studies often include only one isolate per sample, potentially missing a diverse population^7,12^. Sequencing of DNA extracted directly—metagenomics—can describe the diversity of complex microbial communities without a culture step. Several software tools have been recently developed for tracking strains within metagenomes even when they are present at low relative abundance within a complex community^21,22^. However, reconstructing accurate sequences of ARGs and placing them within their genomic context is often challenging due to low sequencing coverage and high heterogeneity, which can confound assembly^23,24^. In addition, ARGs are often carried on plasmids, which makes linking these ARGs to their host organisms challenging^25^. Plate-sweep metagenomics is a recent intermediate approach between isolated colonies and metagenomics that includes a culture step to select for target viable organisms of interest from a sample followed by sequencing the total biomass from the plate. While the overall microbial complexity of a sample is reduced, sequencing effort can still be wasted as most selective media are not species specific^26,27^.

In this Article, we aimed to elucidate bacterial strain-sharing and ARG dissemination between humans, domesticated animals and the household environment. The study area included two low-income informal settlements in Nairobi, Kenya, one of which had access to chlorinated water while the other received untreated water. Given its ubiquity and role as a common human commensal as well as clinically important and often antibiotic-resistant pathogen, we selected *Escherichia coli* as the target organism^28,29^. To capture strain diversity within each sample and achieve high depth of sequencing coverage, we pooled up to five presumptive *E. coli* isolates cultured from the same sample before sequencing. Herein, we call our approach ‘PIC-seq’ (Pooling Isolated Colonies for sequencing). Leveraging matched sample sets of human stool, poultry cloacal swabs, canine faeces, stored drinking water and soil from 50 households, we used PIC-seq to determine strain-sharing patterns. In addition, we reconstructed the resistomes of pooled *E. coli* isolates at the sample level to explore the role of strain dissemination versus horizontal gene transfer in the spread of ARGs.

## Results

### Household characteristics

**Extended Data Fig. 1:**
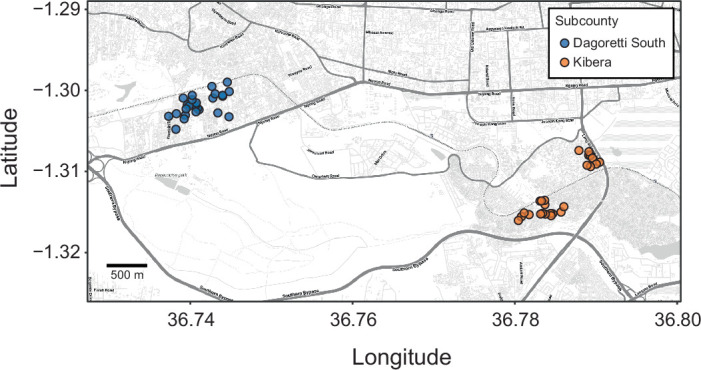
Location of enrolled households across Dagoretti South and Kibera in Nairobi, Kenya. Households in Kibera had access to piped chlorinated water from communal taps, whereas Dagoretti South did not have access to chlorinated water.

We enrolled poultry-owning households (*n* = 50) with at least one child under the age of 5 years across two subcounties (Dagoretti South, *n* = 25 and Kibera, *n* = 25) in Nairobi, Kenya, as part of a cross-sectional study (Extended Data Fig. 1). Urban informal settlements in Kenya are often organized into compounds consisting of households sharing a common courtyard. We collected poultry, canine, soil and water samples from each household on the first visit; 7 days later, we collected human stool samples from the same households, including 52 children under the age of 5 years, 34 siblings (up to 15 years old), and 46 mothers (Extended Data Table 1). The median household size was 5 (interquartile range, 4–6 members), the sex of enrolled children was evenly distributed across subcounties. Households from Dagoretti South and Kibera had similar wealth indices, as determined by household assets including availability of electricity, TV, mobile phone and a stove (Supplementary Table 1). The purpose for poultry ownership across both subcounties was primarily nutrient provision (via meat and eggs) or income generation (Supplementary Table 2). Only four households reported using antibiotics for poultry rearing. Over half (56%; 28/50 households) of respondents reported allowing poultry and canines into the household.

Enrolled households collected drinking water from 37 communal taps. *E. coli* contamination at the source was rare; only two (5.4%; 2/37) of water samples from community taps tested positive (Supplementary Table 3). In Kibera, all households collected drinking water from chlorinated piped water, as confirmed by chlorine residual testing at the tap. By contrast, water sources in Dagoretti South varied, with households collecting water from boreholes (44%; 11/25 households), piped water (44%; 11/25 households) and water tanks (12%; 3/25 households), providing unchlorinated water (Supplementary Table 3). Additional household-level water treatment of stored water was slightly more common in Kibera (24%; 6/25 households) than in Dagoretti South (12%; 3/25 households). Most households stored their water in fully covered containers (82%; 41/50 households), primarily in jerry cans or plastic buckets. Water extraction methods included pouring directly from containers (58%; 29/50 households) or by dipping containers or glass cups into them (30%; 15/50 households). Stored water was intended for human use and generally not provided to animals.

### Strain diversity varies by sample type

**Fig. 1:**
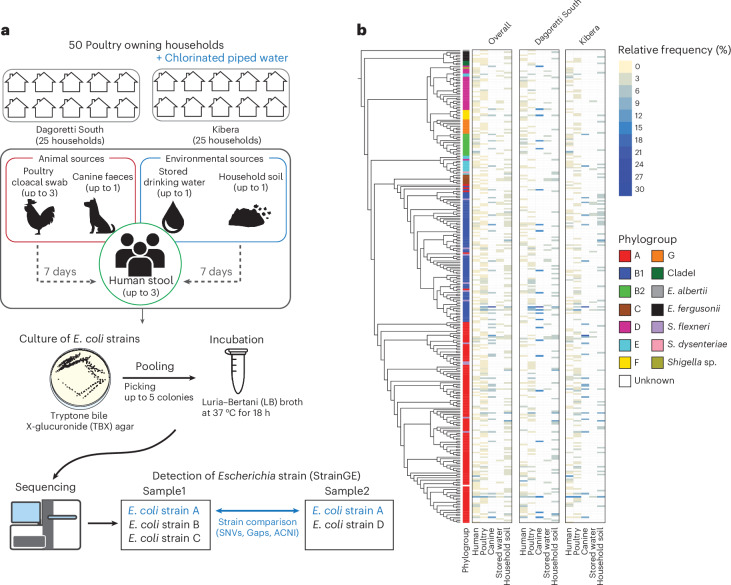
Overview of study design and StrainGE-detected *E. coli* strain across sample types. **a**, Schematic of sample collection and pooling *E. coli* isolates cultured for sequencing. **b**, Cladogram of all isolates built using maximum likelihood based on the alignment of core genes from genomes of reference strains with 1,000 bootstraps. The heat map shows relative frequency of reference strain detection in each sample type, either from overall households or from households within each subcounty. Icons representing sample types and incubation tubes are sourced from The Noun Project under a royalty-free license. Source data

A total of 321 samples (132 human stool, 111 poultry cloacal swabs, 17 canine faeces, 22 stored drinking water and 39 household soil) were collected at the 50 enrolled households. From each sample, up to 5 isolates were pooled together for PIC-seq, capturing a total of 1,516 colonies. Each sample pool was sequenced and analysed with StrainGE v1.3.3 (ref. ^21^) to estimate the number of strains present in each sample, and their corresponding representative genomes, using a database of 2,496 *Escherichia* and *Shigella* reference genomes, clustered at 99% k-mer similarity (Methods). We identified 800 total strains that corresponded to 299 unique representative reference strains (Fig. 1a). The identified reference strains encompassed 279 *E. coli*, 1 *Escherichia albertii*, 6 *Escherichia fergusonii*, and 13 strains within the genus *Shigella* (Fig. 1b). Out of the 13 identified *Shigella* strains, 11 were assigned to *Shigella flexneri*, and the remaining two were assigned to *Shigella dysenteriae* and *Shigella* sp.

**Extended Data Fig. 2:**
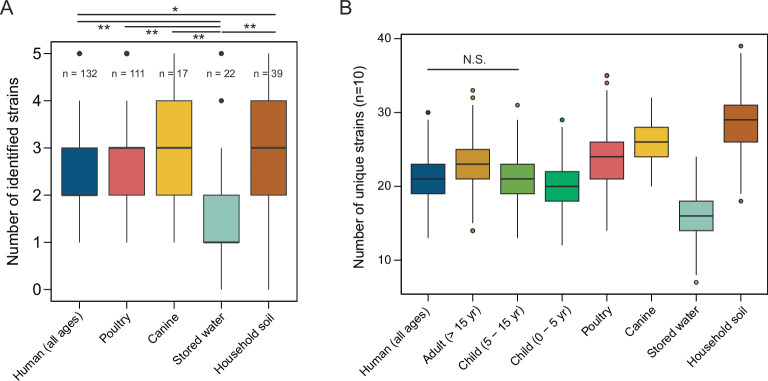
Comparison of the number of detected E. coli strains by sample type. (**A**) Number of strains identified by StrainGE in each sample across sample types. Bars marked with asterisks denote statistically significant differences (*: adjusted *p* < 0.05; **: adjusted *p* < 0.01). (**B**) The number of unique strains identified in subsampled sets (*n* = 10) through 1,000 iterations, categorized by host type. The box plot shows the median (middle line), interquartile range (Q1–Q3), and whiskers extending to 1.5× IQR; points beyond whiskers are outliers. Bars marked with N.S. indicate no significant difference, while others show significant differences. All statistical tests were performed using the two-sided Mann-Whitney U Test (adjusted *p* < 0.05). Source data

Stored drinking water had significantly lower counts of identified strains per sample compared to other sample types, and household soil had significantly higher strain counts per sample than human stool (Extended Data Fig. 2a); no other significant differences were observed. We then investigated how reference strains are distributed within sample types. To account for different sample sizes of each sample type, we subsampled an equal number (*n* = 10) of samples with 1,000 iterations and compared the number of unique reference strains (Extended Data Fig. 2b). Of all sample types, household soil samples exhibited the highest number of unique reference strains, while stored drinking water samples showed the lowest. The number of unique reference strains from poultry cloacal swabs and canine faeces was greater than from human stool samples. In human stool, the number of unique reference strains increased with age (Extended Data Fig. 2b).

**Extended Data Fig. 3:**
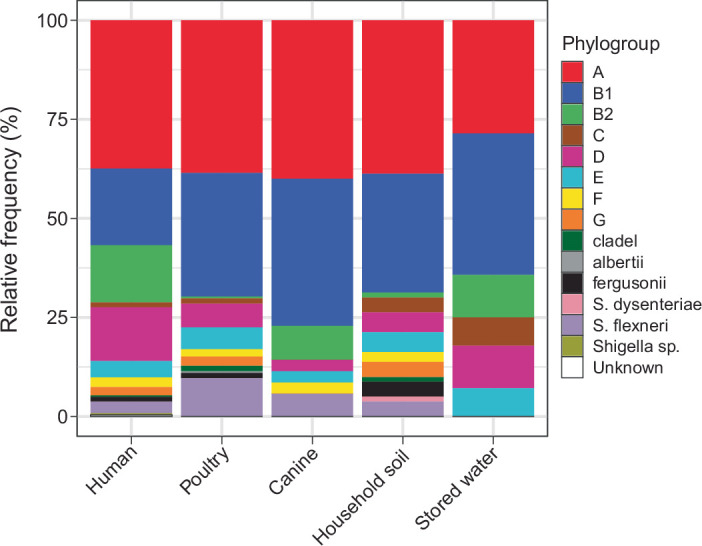
Phylogroup composition of identified representative reference strains by StrainGE, across sample types. The total number of reference strains detected in each sample type was as follows: human (308 reference strains), poultry (293), canine (48), stored water (35) and household soil (116). Source data

**Extended Data Fig. 4:**
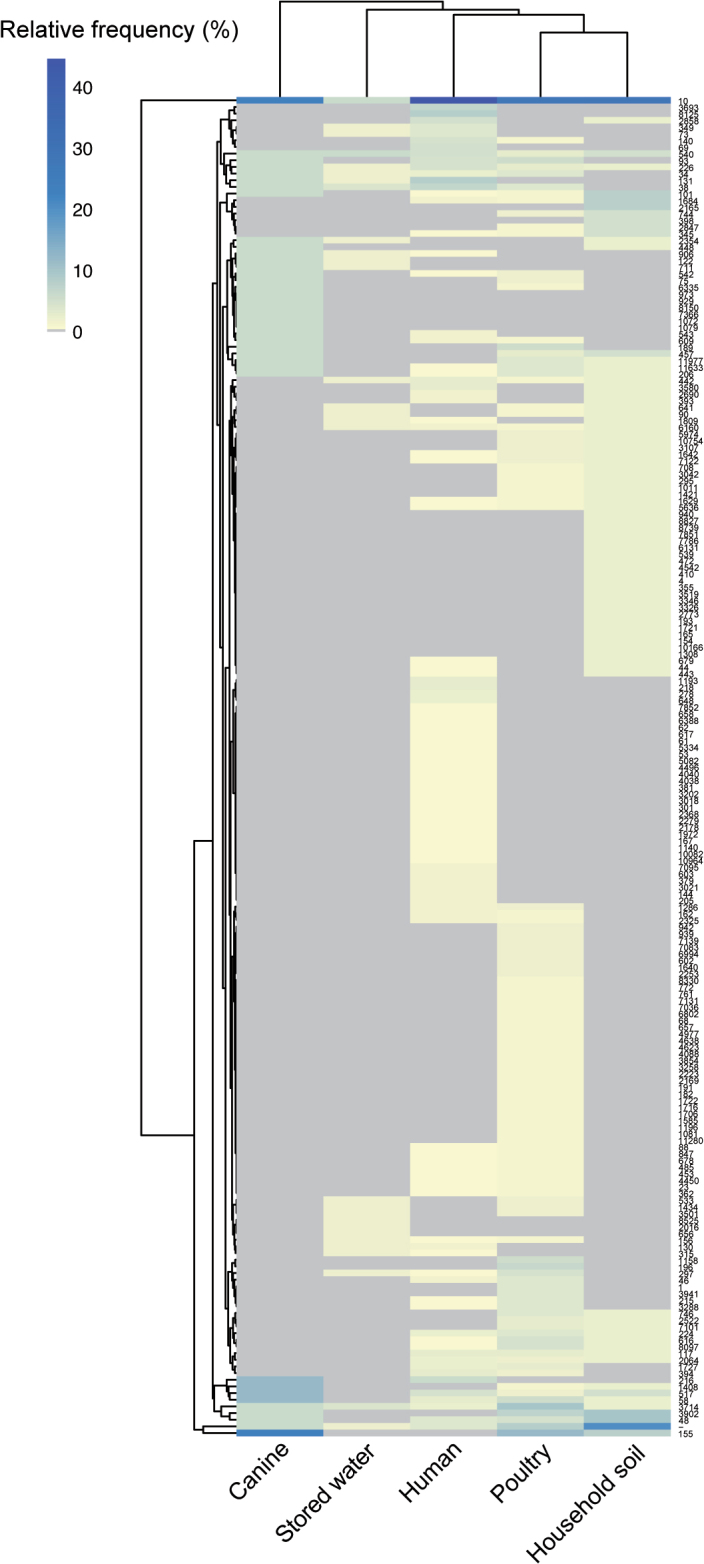
Relative frequency of multilocus sequence types (MLSTs) of StrainGE-identified reference strains in each sample type. Hierarchical clustering was applied to both rows and columns using Euclidean distance and complete linkage, grouping similar STs and samples based on frequency patterns. Color intensity represents relative frequency, with missing values shown in grey. Source data

We compared the phylogroup compositions of the representative reference *E. coli* strains identified across sample types. Phylogroup A was the most abundant with 31.4% to 46.4% of all identified strains across all sample types (Extended Data Fig. 3). The next most common phylogroup was B1, although its frequency among strains identified from humans was noticeably lower (17.2%) than in other sample types (30.4–37.1%). Phylogroups B2 and D were found to be enriched in humans (phylogroup B2, 12.3%; phylogroup D, 11.7%), stored drinking water (phylogroup B2, 8.6%; phylogroup D, 11.4%) and B2 in canine samples (6.3%). In addition, *S. flexneri* was more frequently found among the strains from poultry (7.5%) and canine (4.2%) samples, compared to other sample types. A total of 200 unique multilocus sequence types (MLST) were identified, with ST10 being the most frequently detected across all sample types (Extended Data Fig. 4).

### Strains included *E. coli* pathotypes and *Shigella*

**Fig. 2:**
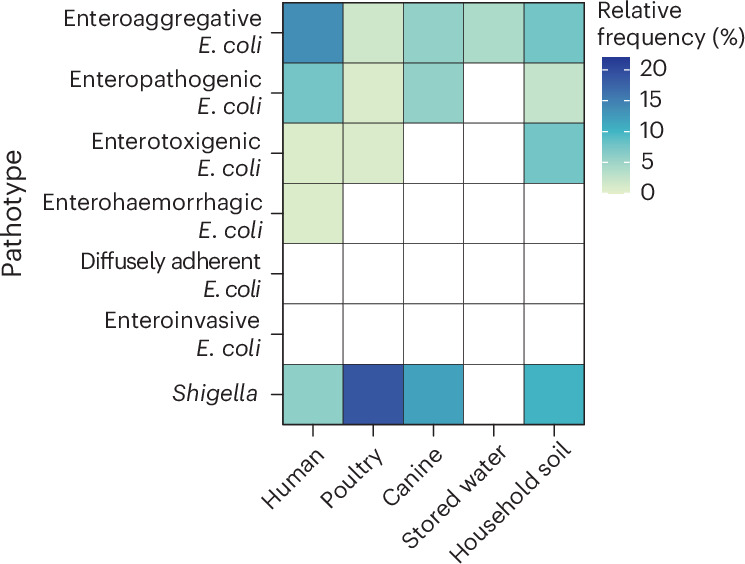
Prevalence of *E. coli* pathotypes and *Shigella* strains across sample types. Detection of *E. coli* pathotypes based on specific sets of virulence genes associated with each pathotype. The presence of *Shigella* was identified by StrainGE. Source data

To examine the prevalence of *E. coli* pathotypes across sample types, we investigated the presence of known virulence genes on the assembled contigs for each sample (Methods). Humans carried the most diverse pathotypes of *E. coli*, with enteroaggregative *E. coli* (EAEC) (13.6% of human samples) and enteropathogenic *E. coli* (EPEC) (7.6%) most frequently detected (Fig. 2). EAEC and EPEC were also frequently detected in canine faeces (EAEC, 5.9% and EPEC, 5.9% of canine samples). In poultry cloacal swabs, EAEC (1.8% of poultry samples), EPEC (0.9%) and enterotoxigenic *E. coli* (ETEC) (0.9%) were detected. In stored drinking water, EAEC (4.1% of drinking water samples) was the only pathotype detected, while household soil had more diverse pathotypes present including EAEC (7.7% of soil samples), ETEC (7.7%) and EPEC (2.6%). Diffusely adherent *E. coli* and enteroinvasive *E. coli* were not detected in any of the samples. *Shigella* was highest in poultry cloacal swabs (19.0% of poultry samples), followed by canine faeces (11.8% of canine samples), household soil (10.3% of soil samples) and human stool (6.1% of human samples) (Fig. 2). No *Shigella* strains were detected in stored drinking water.

### Human-environment strain-sharing is frequent within households

**Fig. 3:**
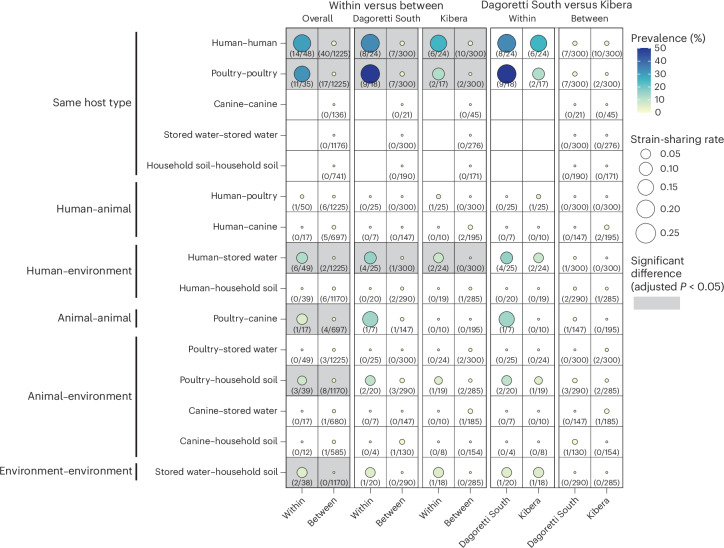
Strain-sharing patterns within and between households. The size of each circle corresponds to the strain-sharing rate (average strain-sharing events/total possible pairs in each household or household pair). The colour gradient of each circle represents the prevalence of strain-sharing, calculated as the percentage of households or pairs of households where strain-sharing was detected relative to the total number of households or household pairs, with actual numbers provided as texts below each circle. Two-sided permutation tests with 1,000 bootstraps were used for testing for significant differences between mean strain-sharing rates, and all resulting *P* values were adjusted using Benjamini–Hochberg correction. Source data

We examined strain-sharing patterns across humans, poultry, canine, soil and stored drinking water within and between households. Sample pairs with strains with over 99.95% average callable nucleotide identity (ACNI) by StrainGE were considered to share strains based on previous benchmarking^21^. We identified 154 strain-sharing sample pairs and compared the average strain-sharing rates (the average strain-sharing events/total possible pairs in each household or household pair) within or between households across different sample type pairs. Within households, strain-sharing was most frequent between the same host type (human–human rate, 0.19 cases per sample pair; poultry–poultry rate, 0.15 per sample pair) with rates significantly higher than the rates between households (human–human rate, 0.0051, *P* < 0.001; poultry–poultry rate, 0.0023, *P* < 0.001) (Fig. 3). Strain-sharing between animals (poultry and canines) was also significantly higher within households (rate, 0.059) compared to between households (rate, 0.0018, *P* = 0.006). Direct human–animal strain-sharing was nearly absent; a strain shared between human and poultry was detected in just one household, with the rates observed between households not significantly different from within households. Strain-sharing with environmental sources (human–water rate, 0.065; poultry–soil rate, 0.038) was frequently observed within households and at significantly higher rates than between households (human–water rate, 0.00030, *P* < 0.001; poultry–soil rate, 0.0024, *P* = 0.0048). In addition, the strain-sharing rate between stored drinking water and soil within households (rate, 0.053) was significantly higher than between households, where no strain-sharing was detected (*P* < 0.001). Unlike within households, where human–water strain-sharing was most common (and significantly higher than human–animal sharing, *P* = 0.027), human–water strain-sharing was one of the lowest between households (rate, 0.00030) (Fig. 3 and Supplementary Table 4).

### Contaminated drinking water facilitates human–human strain-sharing

**Fig. 4:**
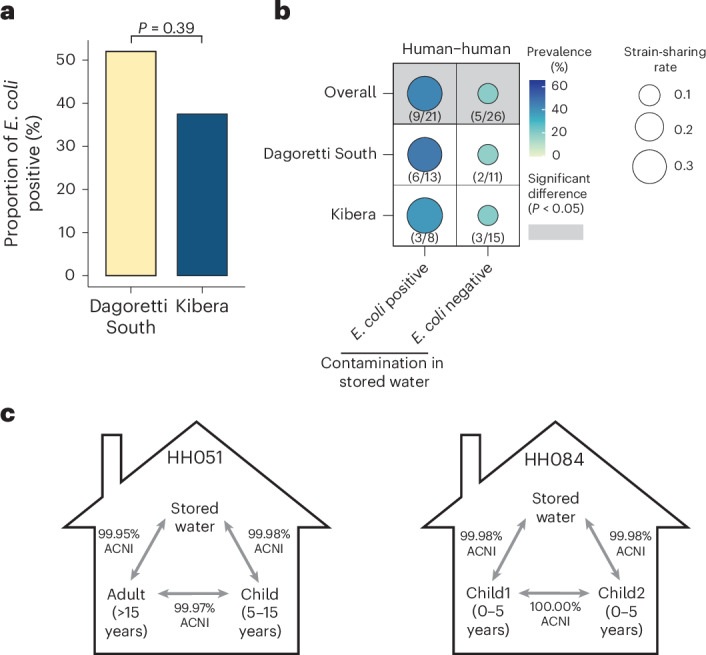
Strain-sharing within households stratified by the presence of *E. coli* contamination in stored drinking water. **a**, Proportion of households with *E. coli*-contaminated stored water in Dagoretti South and Kibera, with the difference tested using two-sided Fisher’s exact test. **b**, Comparison of human–human strain-sharing within households with and without *E. coli* contamination in stored drinking water; two-sided permutation tests with 1,000 bootstraps were used to test for significant differences between mean strain-sharing rates. *P* values are unadjusted. **c**, Humans and stored drinking water share the same strain within two households. Source data

In Kibera, households had access to piped chlorinated drinking water. We observed poorer water quality in Dagoretti South; 52.0% of households (13 out of 25) in Dagoretti South had *E. coli*-contaminated stored drinking water compared to 37.5% of households in Kibera (9 out of 24) (Fig. 4a). We hypothesized that chlorinated water could reduce human–water strain-sharing within households. Indeed, we observed less human–water strain-sharing in Kibera (rate, 0.049) than Dagoretti South (rate, 0.080), although the difference was not statistically significant (Fig. 3). Given that humans within households share the same drinking water source, contaminated stored drinking water may play a role in strain-sharing between humans within households. Indeed, human–human strain-sharing was observed in 42.9% (9/21) of households with *E. coli*-contaminated stored drinking water, compared to just 19.2% (5/26) of households without *E. coli* contaminated water (Fig. 4b). The human–human strain-sharing (rate, 0.32) within households with *E. coli*-contaminated stored water was significantly higher than in households with clean water (rate, 0.090, *P* = 0.026) (Fig. 4b). No other strain-sharing sample type pairings showed significant differences when households were stratified by stored water contamination (Supplementary Table 5). Furthermore, we observed identical strains shared across two humans and water in two households (household ID, 051 in Kibera and 084 in Dagoretti South), lending evidence for strain-sharing between humans through consumption of contaminated stored drinking water (Fig. 4c).

### PIC-seq outperforms metagenomics

**Extended Data Fig. 5:**
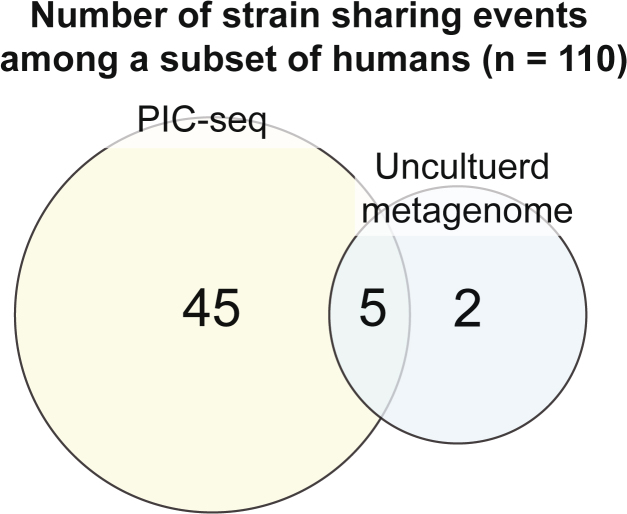
Comparison of strain-sharing events between PIC-seq in this study and uncultured metagenome sequencing froma subset of matching samples. *E. coli* strain-sharing results from PIC-seq were compared to those obtained from metagenomic sequencing of matched human stool samples (*n* = 110) without culture enrichment.

We evaluated the performance of PIC-seq in investigating *E. coli* strain-sharing patterns by comparing the results with metagenome sequence data generated without the culture enrichment step. As part of another study, total DNA was extracted from a subset of matched human stool samples in this study (110 samples) and sequenced. StrainGE detected a slightly lower number of strains across the total metagenome samples (230 strains) versus the corresponding PIC-seq samples (248 strains). Furthermore, while the PIC-seq approach detected strain-sharing in 50 sample pairs, strain-sharing was detected in only 7 metagenomic sample pairs (5 pairs overlapping between the two approaches), possibly due to insufficient coverage depth for detecting genomic substitutes in *E. coli* between samples (Extended Data Fig. 5).

### Distribution of clinically relevant ARGs

To investigate ARG transmission, we examined the distribution and diversity of ARG variants based on the assembled contigs for each sample type. To assess whether long-read sequencing provided higher resolution insights into ARG transmission, we benchmarked using simulated pooled *E. coli* datasets on contigs assembled from Oxford Nanopore Technology long reads versus Illumina short reads. While long read data consistently generated longer contigs than those from short reads across all simulated datasets—in terms of the proportion of the recovered genome fraction, the length of the largest alignment and N50—they exhibited the lowest accuracy in their sequence identity to the original genomes (Supplementary Fig. 1). Hybrid assembly using both short and long reads marginally improved contig length but resulted in lower accuracy compared to short-read assemblies. Thus, we opted to analyse ARGs using only contigs assembled from short reads. In addition, we benchmarked our assembly approach, which uses reference-based binning before de novo assembly to reconstruct resistomes, against sole de novo assembly. Our approach significantly outperformed sole de novo assembly, reconstructing 81.6% to 89.2% of the original resistome compared to just 15.1% to 27.8% (Supplementary Fig. 2).

**Extended Data Fig. 6:**
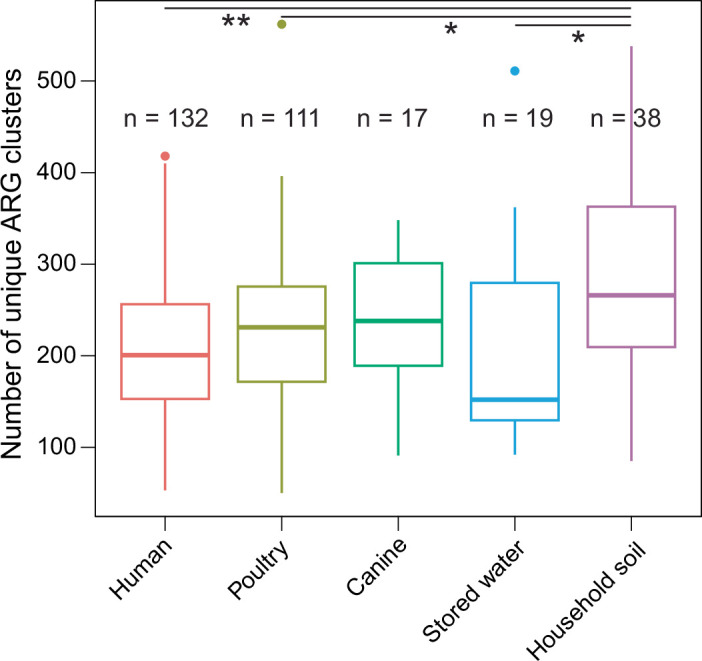
Number of unique ARG clusters in each sample across sample types. The box plot shows the median (middle line), interquartile range (Q1–Q3), and whiskers extending to 1.5× IQR; points beyond whiskers are outliers. Bars marked with asterisks denote statistically significant differences as determined by two-sided Mann-Whitney U Test (*: adjusted *p* < 0.05; **: adjusted *p* < 0.01). Three stored water samples and one household soil sample without any StrainGE *E. coli* strain detection were excluded from this comparison. Source data

We grouped all identified ARGs on the contigs into clusters based on their nucleotide sequences at 100% identity and compared the number of unique ARG clusters observed in each sample type. Similar to strain diversity patterns, household soil had the highest number of unique ARG clusters (median, 266) and stored drinking water the lowest (median, 152) (Extended Data Fig. 6). No significant difference was observed between humans (median, 200.5) and animals (poultry median, 231; canine median, 238).

**Fig. 5:**
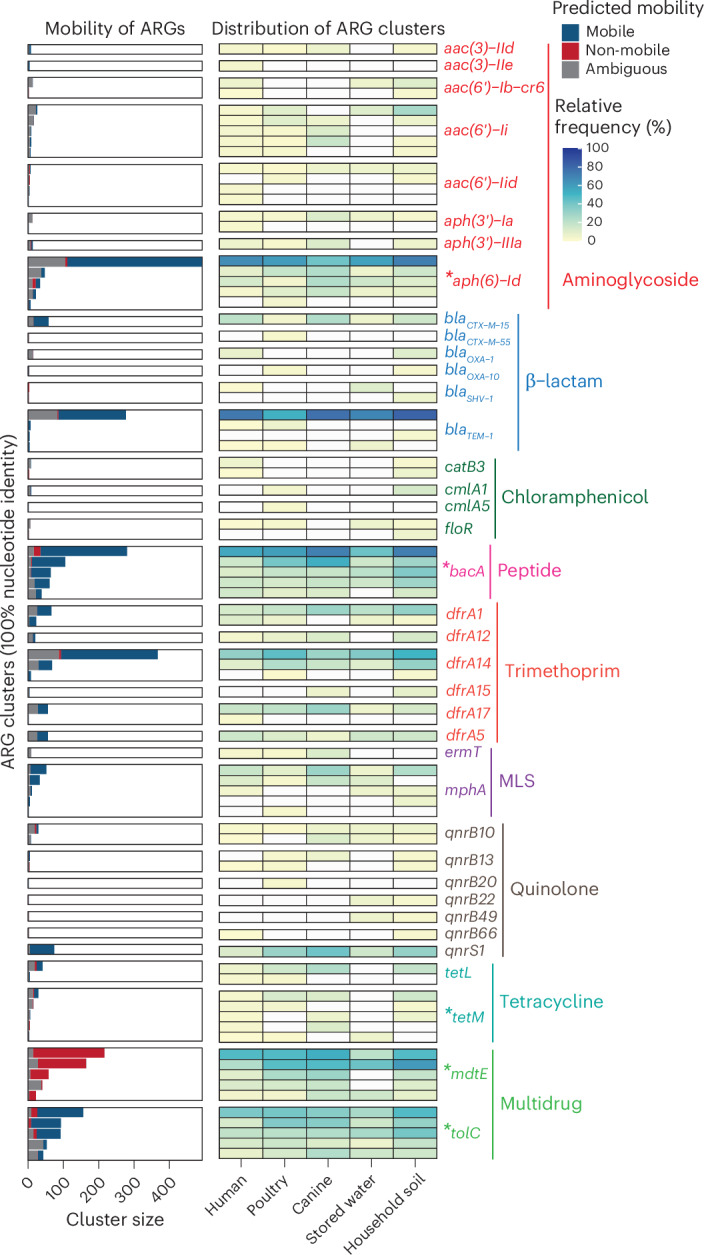
Distribution and mobility of clinically relevant ARG clusters at high risk (Rank I) identified across humans, animals and the environment. *Asterisks on ARG annotations indicate that only the top five most abundant ARG clusters are depicted in the figure. Source data

**Extended Data Fig. 7:**
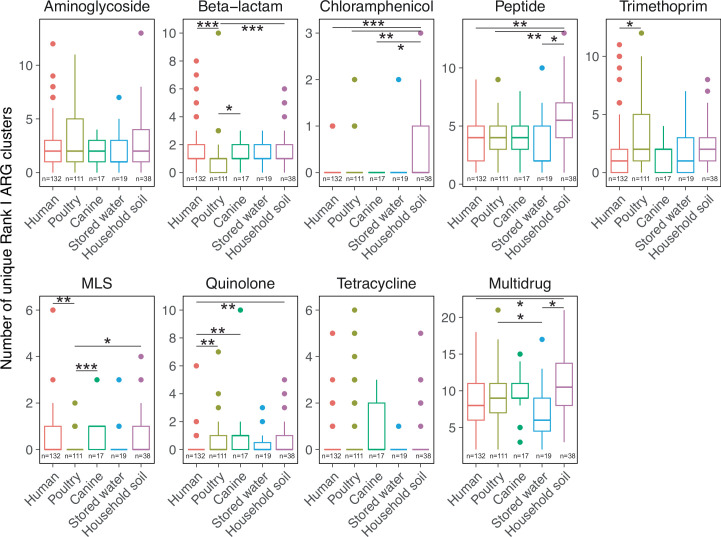
Number of Rank I unique ARG clusters in each sample across sample types grouped by drug classes. The box plot shows the median (middle line), interquartile range (Q1–Q3), and whiskers extending to 1.5× IQR; points beyond whiskers are outliers. Bars marked with asterisks denote statistically significant differences as determined by two-sided Mann-Whitney U Test (*: adjusted *p* < 0.05; **: adjusted *p* < 0.01; ***: adjusted *p* < 0.001). Three stored water samples and one household soil sample without any StrainGE *E. coli* strain detection were excluded from this comparison. Source data

We investigated the diversity and distribution of high-risk ARGs, classified as Rank I ARGs by Zhang et al.^30^, across our sample types. Rank I ARGs refer to gene families that have been reported to be significantly enriched in human-associated environments, are associated with gene mobility and are known to be present in ESKAPE pathogens (*Enterococcus faecium*, *Staphylococcus aureus*, *Klebsiella pneumoniae*, *Acinetobacter baumannii*, *Pseudomonas aeruginosa*, and *Enterobacter* sp.). Humans had significantly higher numbers of Rank I ARG clusters conferring resistance to beta-lactam and macrolide–lincosamide–streptogramin drug classes than poultry, while Rank I ARG clusters of trimethoprim and quinolone were found to be higher in poultry (Extended Data Fig. 7). We assessed ARG mobility in our samples based on adjacency to mobile genetic elements (MGEs). For ARGs where mobility could be determined, an average of 64.2% of ARGs within Rank I ARG clusters were predicted to be mobile, except for *mdtE*, an efflux pump gene associated with multidrug resistance, for which none were predicted to be mobile (Fig. 5 and Supplementary Table 6). Prevalence of ARG clusters across humans, animals and the environment was similar, with several ARG clusters prevalent in all sample types. *aph(6)-Id*, which confers resistance to the antibiotic class of aminoglycoside, was the most frequently present in all groups, including in 41.2% of canine samples. Among β-lactamase genes, a *bla*_TEM-1_ cluster was most prevalent, with detection in 51.4% of poultry samples. *bla*_CTX-M-15_, an extended-spectrum β-lactamase (ESBL) that confers resistance to third-generation cephalosporins (Fig. 5), was present across all sample types at 20.5% in humans, 23.5% in canines, 15.8% in household soil, 4.5% in poultry and 5.3% in stored water.

### Strain-sharing disseminates ARGs

**Extended Data Fig. 8:**
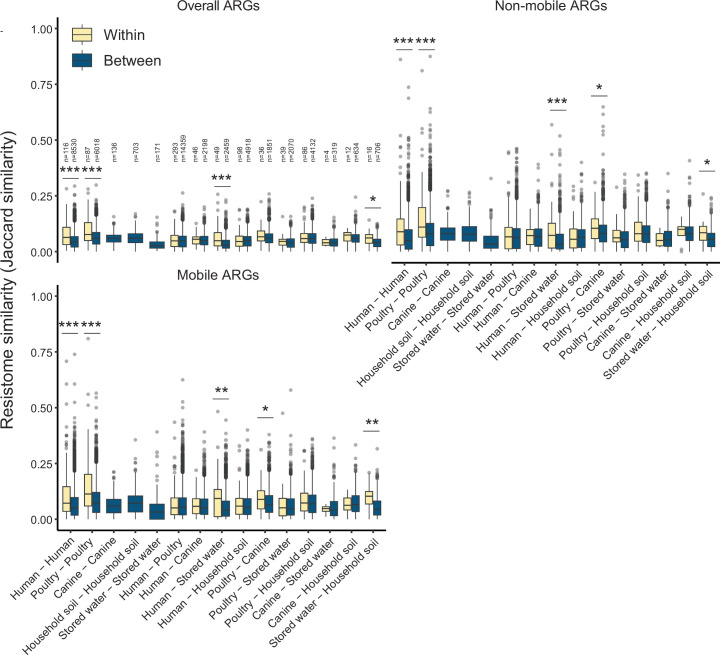
Comparison of overall, non-mobile, and mobile resistome similarities within versus between households calculated based on Jaccard similarities. The box plot shows the median (middle line), interquartile range (Q1–Q3), and whiskers extending to 1.5× IQR; points beyond whiskers are outliers. The asterisk denotes statistically significant differences as determined by two-sided Mann-Whitney U Test (*: adjusted *p* < 0.05; **: adjusted *p* < 0.01; ***: adjusted *p* < 0.001). Source data

To investigate whether spatial proximity is associated with similarity in ARG profiles, sample resistomes were compared within versus between households. Resistome similarity closely mirrored strain-sharing rates, with significantly higher resistome similarity within households compared to between households for human–human, poultry–poultry, human–water and water–soil (Extended Data Fig. 8). In addition, poultry–canine pairs shared significantly higher resistome similarity within households than between households when stratified by predicted mobility (Extended Data Fig. 8).

**Fig. 6:**
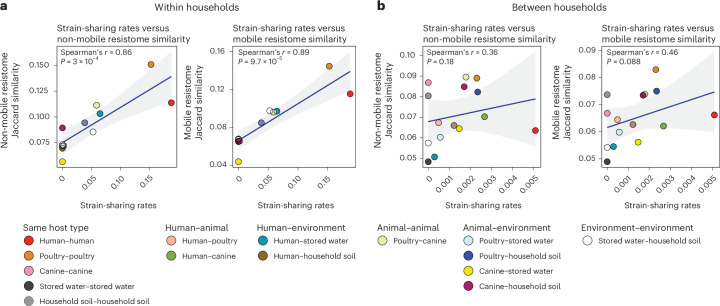
Spearman’s correlation analyses of non-mobile and mobile resistome similarity patterns with strain-sharing patterns. **a**,**b**, The analyses within (**a**) and between (**b**) households. Spearman correlation coefficients and *P* values (two-sided test) are shown in each panel. Grey error bands indicate 95% confidence interval of the fitted Spearman correlation trend. Source data

**Extended Data Fig. 9:**
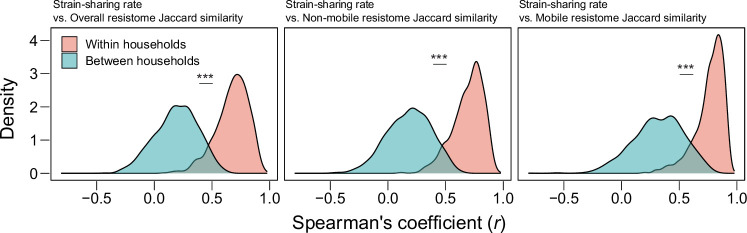
Distribution of Spearman’s coefficient (r) between resistome similarity and strain-sharing rate. The coefficients were calculated based on samples from resampled households bootstrapped 1000 iterations. The asterisks (***) indicate a significant difference between the distributions (two-sided Mann-Whitney U test, *p* < 0.001). Source data

To further investigate the influence of strain-sharing on resistome composition, we performed a correlation analysis between mean strain-sharing rates and mean resistome similarities between sample types (Fig. 6). We hypothesized that if ARGs are not specific to certain strains due to frequent horizontal gene transfer and are already prevalent among samples, a weak correlation between strain-sharing rates and resistome similarities would be expected. Both non-mobile and mobile resistome similarities were strongly correlated with strain-sharing rates within households (non-mobile versus strain-sharing rate, Spearman’s *r* = 0.86; *P* = 0.0003 and mobile versus strain-sharing rate, Spearman’s *r* = 0.89; *P* = 0.000097), suggesting frequent strain-sharing within households was a strong factor for sharing similar resistomes (Fig. 6). However, no significant correlation was observed between households, presumably due to an insufficient number of strain-sharing events to affect mean resistome similarities. A bootstrapped analysis revealed different correlation coefficient distributions within versus between households (Extended Data Fig. 9).

## Discussion

Here we observed high rates of *E. coli* strain-sharing between humans and stored drinking water within households compared to between households, emphasizing the role of drinking water as a key route for bacterial strain exchange with humans^31,32^. *E. coli*-contaminated drinking water was associated with higher human–human strain-sharing rates in households, and we captured instances of the same strain shared between humans and water in the same household. Frequent strain exchange between drinking water and humans could explain the similarities in phylogroup profiles of strains between humans and stored drinking water, including the comparable proportion of phylogroups B2 and D^33^. Our results are consistent with bacterial strains ingested through water consumption readily colonizing the human gastrointestinal tract^34,35^.

We observed lower levels of human–water strain-sharing rates and stored drinking water *E. coli* contamination in Kibera compared to Dagoretti South, suggesting access to chlorinated water in Kibera disrupted strain-sharing via drinking water^36^. Water quality data collected by the parent study documented significantly lower levels of stored drinking water *E. coli* contamination in Kibera (22.6%) compared to Dagoretti South (68.2%) with a larger set of households (120 households) (Fisher’s exact test; *P* < 0.001)^37^. A previous randomized controlled trial of community-wide passive chlorination of drinking water in urban Bangladesh found a 23% reduction in 7 day child diarrhoea prevalence, suggesting the effectiveness of chlorinated water in disrupting bacterial transmission^36^. Considering the complex bacterial transmission routes in densely populated urban informal settings^8^, our findings highlight chlorinated drinking water as a key strategy to disrupt household strain-sharing. To ensure its effectiveness, dosing optimal amounts of chlorine with improved safe storage practices (for example, hand hygiene when extracting water and limiting storage duration) is critical^36,38^, as stored water can still become microbially contaminated during prolonged storage due to degradation of free-chlorine levels. Rare *E. coli* contamination in communal source water, coupled with the absence of strain-sharing between stored water from different households, suggests stored water contamination likely occurred after collection in this study.

Our results are consistent with clonal spread of bacteria being the main driver shaping resistomes of pooled *E. coli* strains. ARG variants associated with MGEs have the potential for horizontal gene transfer, which would result in overlapping ARGs between non-identical or phylogenetically distant strains. In our study, the significant correlation of mobile (and non-mobile) ARG similarity with strain-sharing rates between samples within households indicates that close-proximity strain-sharing had a greater influence on resistome similarities than ARG exchange via horizontal gene transfer. This observation aligns with a previous study in Kenya^39^, where the MLST of *E. coli* isolates from humans, domestic animals and wild animals best explained the variance of the mobile ARG assemblages among them. Within households, human–water pairs showed higher resistome similarity (due to clonal transmission) than human–animal pairs, underscoring the role of frequent bacterial strain-sharing in ARG spread. Between households, where strain-sharing events were rare, no correlations were observed between resistome similarities and strain-sharing rates.

Environmental sample resistomes possessed many high-risk ARGs^30^. Rank I ARG variants within environmental samples had similar or even higher diversity than human and animal hosts across antibiotics classes. Although clinically relevant low-abundance strains, such as ESBL-producing *E. coli*, may have been underestimated due to not targeting specific antibiotic resistance phenotypes for PIC-seq in our study, ARGs such as *bla*_CTX-M-15_, conferring resistance to third-generation cephalosporins, were detected consistently across all sample types. This aligns with findings from a Malawi study^40^, which reported the widespread presence of ESBL-producing *Enterobacterales* across humans, animals and the environment, particularly during the wet season. Although the use of antibiotics for poultry was rare in our study, the widespread use of quinolones and trimethoprim by poultry farmers in Nairobi, Kenya^41–43^, coupled with the community-scale strain-sharing, could explain the higher number of Rank I ARG clusters observed in poultry compared to humans.

Our results demonstrate the value of PIC-seq for examining strain-sharing and the prevalence of ARGs. Unlike the conventional single-isolate sequencing approach, which requires either a larger sample size or a focus on specific strain phenotypes to improve the likelihood of detecting strain-sharing, PIC-seq efficiently identified meaningful strain-sharing patterns with fewer samples (1,516 colonies from 321 samples), enabling its use in resource-constrained settings. PIC-seq showed higher performance in capturing more strain-sharing events (50 sharing events) compared to those identified through traditional metagenomic sequencing (only 7 sharing events) in a subset of matching stool metagenomes sequenced for another study (Extended Data Fig. 5). Furthermore, our integration of additional de novo assembly with reference-based binning assembly enabled the recovery of ARGs located in genomic regions of pooled organisms that differed from reference genomes, outperforming sole de novo assembly (Supplementary Fig. 3). ARGs recovered from unbinned reads were mostly associated with MGEs, such as plasmids and genomic islands, which can circulate among strains via horizontal gene transfer^44^. While these ARGs could not be tied to a specific strain, we were able to better capture the resistome of pooled isolates from each sample. Compared to sequencing of single isolates or sequencing the entire microbiome for strain tracking, our approach offers a distinct advantage in that it allows for simultaneous capture of strain identity and sensitive detection of ARGs with genomic context, although its application is limited to targeted bacterial species. PIC-seq is a scalable protocol for examining strain-sharing patterns that could be replicated for other bacterial species.

## Methods

### Study sites and sample collection

To investigate *E. coli* strain-sharing between humans, domesticated animals and the environment, 50 poultry-owning households with at least one child under the age of 5 years were enrolled in two subcounties, Dagoretti South (*n* = 25) and Kibera (*n* = 25), in Nairobi, Kenya, during June to August 2019 as part of a cross-sectional study. One household was randomly enrolled from each compound that owned poultry. Written informed consent was obtained from each adult participant before stool sampling, and child assent and parental written consent were obtained for each child participant. Participants received a pack of soap as compensation. A household survey was performed along with the sample collection to collect demographic information and household characteristics including water, sanitation, and hygiene access and practices. No statistical methods were used to pre-determine sample sizes, but our sample sizes are similar to those reported in previous publications^12^. Data collection and analysis were not performed blind to the conditions of the experiments.

Each household was visited twice for the sample collection. During the first visit, we collected up to three poultry cloacal swabs (depending on the number of poultry present), one canine faecal sample, one household soil and one stored drinking water sample from each household. A trained veterinary student administered the poultry cloacal swabs and placed them in storage tubes filled with Cary–Blair transport medium. To collect canine faeces, the top layer of fresh faeces from the centre of the pile was transferred into a 50 ml centrifuge tube with a sterile plastic scoop. For household soil collection, a 30 cm × 50 cm square area closest to the entrance of the household was marked, and the top layer of the entire surface of the marked area was scraped with a sterile disposable plastic scoop. Approximately 150 g of soil was collected and transferred into a 500 ml sterile Whirlpak bag. The stored water within each household was collected by withdrawing 350 ml of the water from the bottom of the storage container with a sterile serological pipette. The water was then transferred into a 500 ml sterile Whirlpak bag containing 10 mg of sodium thiosulfate. All samples were immediately placed in a cooler filled with ice and transported to the Kenya Medical Research Institute (KEMRI).

To capture transmission of bacterial strains from the environment to humans, approximately 1 week after the initial environmental sampling visit, households were revisited to collect human stool from one household member in the following three age groups: child aged 0–4 years, child aged 5 14 years and adult aged 15 years or older. A stool collection kit was provided during the first visit, which included a 50 ml plastic pot with a sterile scoop for each member with instructions on how to collect the sample. Caretakers were instructed to collect faeces on aluminium foil, then, using sterile gloves and scoops, to transfer the faeces to the plastic pot for collection. The primary caretaker of each household was informed by mobile phone 1 day before the revisit to collect stool from the previous night or the morning of the revisit day. If stool samples were not available on the visit day, households were revisited up to three times to collect the remaining stool samples. After pick-up, the received stool samples were placed in a cooler filled with ice and transported to KEMRI.

The study received ethical approval from the KEMRI Scientific and Ethics Review Unit (12/3823) and the Tufts Health Sciences Institutional Review Board (13205). In addition, a research permit was granted by the Kenyan National Commission for Science, Technology, and Innovation.

### PIC-seq

Here we describe our method of pooling presumptive isolated *E. coli* colonies for sequencing (PIC-seq). *E. coli* colonies were first cultured from the collected samples within 6 h of sample collection. A sterile swab was inserted into 1 g of each collected human stool and canine faeces sample, and the swab was streaked on Tryptone Bile X-glucuronide (TBX) agar plates. Poultry cloacal swabs were directly streaked on the agar plates. Household soil and stored water samples were membrane filtered and cultured on TBX. Collected soil was first screened through 2 mm pore mesh sieves to remove rocks and leaves, then 5 g was homogenized with 50 ml of distilled water. After allowing soil particles to settle for 1 min, 100 μl of supernatant was diluted with 10 ml of distilled water, vacuumed through a 0.45 μm membrane filter and plated. About 100 ml of stored water was directly filtered, then plated on TBX. All plates were incubated overnight (18–24 h) at 44 °C.

After incubation, up to five presumptive *E. coli* colonies (blue-green colour) were selected from each agar plate for pooling. The selected colonies from each plate were transferred into a single 2 ml tube containing 1 ml of Luria–Bertani medium. The pooled *E. coli* colony cultures were incubated overnight (18 h) at 37 °C with shaking at 200 r.p.m. After incubation, 100 μl of the cultures was mixed with 1 ml of 30% glycerol–70% tryptone soy broth solution and stored at −80 °C. The samples were then shipped on dry ice to Tuft University.

Pooled isolates were thawed on ice and inoculated into 5 ml of fresh tryptone soy broth medium with a sterile inoculating loop. The cultures were incubated overnight at 37 °C with 200 r.p.m. shaking, then 1.5 ml of the culture was pelleted by centrifugation at 5,000 × *g* for 10 min. The DNA was then extracted from pellets using DNeasy PowerSoil Pro Kit (Qiagen) following the protocol provided by the manufacturer. The concentration and purity of the extracted DNA were measured by a Qubit 4.0 Fluorometer (Thermo Fisher Scientific), a sequencing library was prepared using Nextera XT kit (Illumina), and shotgun sequencing was performed on Illumina NovaSeq 6000 (Illumina) with a target sequencing depth of 1.5 Gb at the Broad Institute.

### Identification of *E. coli* strains and calculation of strain-sharing rates

The quality of raw sequence reads was examined using FastQC v0.12.1 (ref. ^45^), and low-quality reads were filtered out using Trimmomatic v0.39 (ref. ^46^) with parameters set to LEADING:3, TRAILING:3, SLIDINGWINDOW:4:15 and MINLEN:70. The subsequent reads were used for the identification and comparison of *Escherichia* strains using the software StrainGE v1.3.3, the software specifically designed for accurate calling and comparison of strains in the complex metagenome data based on k-mer comparison, bypassing assembly process^21^. The database for StrainGE was constructed using the complete genome sequences (2,496 genomes) of the genus of *Escherichia* or *Shigella* downloaded from the National Center for Biotechnology Information (NCBI) RefSeq database on 20 August 2022, by using the built-in database construction pipeline (‘ncbi-genome-download’, ‘kmerize’, ‘kmersim’, ‘cluster’ and ‘createdb’). The reference genomes in the database were clustered if they shared 99% of their k-mer content with a Jaccard similarity over 0.90, and to minimize redundancy, only the representative genome sequences of each cluster were retained, resulting in a final set of 885 genomes.

After strain identification, we used StrainGR, the second module of StrainGE v1.3.3, to call sequence variants among samples^21^. Sequence reads were aligned to the genomes of the identified strains in each sample using bwa mem v0.7.17 with default parameters^47^. For sample pairs with the same reference strain, the average ACNI and gap similarity were calculated based on the callable regions, using ‘call’ and ‘compare’ command in StrainGR^21^. The threshold for defining the same strain was set to >99.95% ACNI.

The strain-sharing rates between sample types were calculated, using the results from StrainGR. Within each household, we normalized the number of sample pairs sharing the same strain by the total number of combinations between the certain sample types. We then computed the average rates based on the rates from all households for comparison. For strain-sharing rates between different households, we normalized the number of sample pairs with the same strain by the total number of possible sample pairs, between all combinations of different households. We then calculated the average rates from these inter-household rates for comparisons between certain types of sample. Stored water samples without *E. coli* contamination were included in calculating water-related total number of possible pairs.

### Phylogeny and phylogroup of detected *E. coli* strains

To infer the phylogenetic relationships among the strains identified from the samples, we used the reference genome sequences included in the StrainGE database. Prokka v1.14.5 was used to annotate genes, with the genus parameter set to *Escherichia*^48^. The core genes that exist in 99% of all reference genomes were identified using Roary v3.13.0 (ref. ^49^). The concatenated core gene alignment was generated using MAFFT v7.508, and SNP-sites v2.5.1 (ref. ^50^) was used to extract the alignment regions with single-nucleotide polymorphisms to reduce the size of the alignment. The reduced alignment was uploaded to CIPRES Science Gateway v3.3 (ref. ^51^) (www.phylo.org), and a maximum likelihood tree was constructed using RAxML v8.2.12 (ref. ^52^) with the nucleotide substitution model set to GTRGAMMA with 500 bootstraps. *E. coli* phylogroup was predicted using ClermonTyping v20.03 (ref. ^53^) with default parameters. MLST of the StrainGE-identified reference strains were assigned using the software mlst v2.22.1 (https://github.com/tseemann/mlst) based on the ecoli_achtman_4 scheme.

### Identification of pathogenic *Escherichia* and *Shigella* strains

The identification of pathogenic *E. coli* strains was inferred based on the presence of pathotype-associated virulence genes detected in the assembled contigs for each sample. These virulence genes were annotated from the contigs using ABRicate v1.0.1, in combination with the Virulence Factor Database (download in May 2023)^54^. The presence of different *E. coli* pathotypes was based on the detection of the following gene sets: *agg*, *aat* and *aai* for EAEC; *eae* and *bfp* for EPEC; *elt* and *est* for ETEC; *stx* for enterohaemorrhagic *E. coli*; *afa* and *drg* for diffusely adherent *E. coli*; and *ipa* for enteroinvasive *E. coli*^55–57^. The presence of *Shigella* was additionally investigated based on StrainGST results.

### Annotations of ARGs and MGEs

The integrated assembly pipeline, which combines reference-based binning and de novo assembly, was used to generate contigs for ARGs and MGEs annotation (Supplementary Fig. 4). Plasmid sequences were assembled from sequencing reads using metaplasmidSPAdes v3.15.4 (ref. ^58^) with default parameters. Subsequently, the original reads were aligned and binned to the set of the assembled plasmid sequences and the reference genomes of strains identified through StrainGST, using mSWEEP v.2.0.0 (ref. ^27^) and mGEMs v.1.3.0 (ref. ^26^) with default parameters. For each reference strain, the reads binned to their genome sequences were individually assembled de novo using metaSPAdes v3.15.4 (ref. ^59^) with default parameters. The sequence reads that did not align to any sequences were separately assembled de novo using metaSPAdes. The gene-coding sequences in the assembled contigs were predicted using Prodigal v2.6.3 (ref. ^60^) with default parameters. The translated amino acid sequences of the predicted gene-coding sequences were annotated using DIAMOND blastp v2.0.14.152 (ref. ^61^) against the Comprehensive Antibiotic Resistance Database (CARD) v3.2.4 (ref. ^62^) with the parameters set to ‘–id 90’, ‘–min-orf 25’ and ‘–query-cover 80’. The annotations with the highest bitscore for each gene-coding sequence were retained for the downstream analysis.

To predict the mobility of ARGs, we examined the presence of any MGEs in the flanking regions of the annotated ARGs. We extracted up to 5,000 base pairs from both upstream and downstream of ARGs on the contigs using a custom bash script. The mobileOG-pl pipeline was then used to annotate MGEs with mobileOG-db release beatrix-1.6 as the reference database^63^, which is a manually curated database that includes sequences from eight different databases (ICEBerg, ACLAME, GutPhage Database, Prokaryotic viral orthologous groups, COMPASS, NCBI Plasmid RefSeq, immedb and ISfinder), along with homologues of the manually curated sequences. ARGs with identified MGEs in their flanking region were presumed to be mobile, while those without MGEs were categorized as non-mobile. In instances where the contig length was insufficient (<5,000 base pairs) to detect MGEs in the flanking region, the mobility of these ARGs was considered ambiguous.

### Preparation of simulated datasets for benchmarking

To benchmark our bioinformatics pipeline, we generated simulated datasets that emulate sequence reads we would expect from PIC-seq on five *E. coli* isolates. The paired-end Illumina sequence reads (2 × 150 bp) and the complete genome sequences of five clinically relevant antibiotic-resistant *E. coli* isolates, possessing either *bla*_*CTX-M-15*_ or *mcr-1* genes along with other ARGs, were provided by the Broad Institute. We characterized each isolate based on their complete genome sequences. The phylogroup of each *E. coli* isolate was predicted using ClermonTyping v20.03 (ref. ^53^) with default parameters. MOB-suite v3.1.0 was used to identify any plasmid contents included in the genome files^64^. The gene coding sequences within genomes were predicted using Prodigal v2.6.3 (ref. ^60^) and were annotated using DIAMOND blastp v2.0.14.152 (ref. ^61^) against the CARD v3.2.4 with parameters set to minimum identity of 90%, minimum query cover 80% and minimum amino acid length of 25. The proximity of MGEs to genes annotated as ARGs was examined in a 5,000 bp region both upstream and downstream of the ARGs, using the mobileOG-pl pipeline and the mobileOG-db release beatrix-1.6 as the reference database^63^.

To generate simulated datasets, we subsampled the sequence reads from each *E. coli* isolate and pooled with 6 different proportions, ranging from equal ratios to 400-fold difference in sequence coverage, targeting a total sequencing depth of 2 Gb using seqtk v1.3 (Supplementary Table 7). After pooling sequences, the quality of the reads was screened using Trimmomatic v0.39 (ref. ^46^) with parameters set to LEADING: 3, TRAILING: 3, SLIDINGWINDOW: 4:15, and MINLEN: 70. The downstream analyses were performed subsequently based on these datasets.

### Comparison of resistomes reconstructed from two different assembly strategies

To evaluate the efficacy of using a reference-based binning approach before de novo assembly in reconstructing resistomes, we compared resistome profiles generated from assemblies with and without the binning process. (1) For assemblies that used the binning approach, the procedure followed was identical to the approach used for real samples. The plasmid-like contigs were first assembled out of initial data, and the initial reads were aligned to these plasmid contigs and the reference genomes of strains predicted by StrainGE^21^ using mSWEEP v2.0.0 (ref. ^27^) and mGEMs v1.3.1 (ref. ^26^) with default parameters. The reads binned to each reference genome were individually de novo assembled using metaSPAdes v3.15.4 (ref. ^59^). The unaligned reads were separately de novo assembled using metaSPAdes. (2) The assembly without binning process was simply performed by running metaSPAdes on the initial simulated dataset with default parameters.

For all contigs assembled from two different approaches, the coding gene sequences were predicted using Prodigal v2.6.3 (ref. ^60^), and the genes were annotated by running DIAMOND blastp v2.0.14.152 (ref. ^61^) against the CARD with the same parameter used for the real samples. The co-localization of MGEs was investigated by annotating coding genes located within 5,000 bp both upstream and downstream of identified ARGs. ARGs that had MGEs in their flanking regions were considered to have the potential for mobility. ARGs with no MGEs in their flanking regions, but whose flanking region were shorter than 5,000 base pairs due to contig size limitations, were categorized as having ambiguous mobility. The coding regions of all ARGs were clustered at 100% nucleotide identity together with ARGs from reference genomes for the comparison of resistomes.

### Assembly using short or long sequence reads of simulated datasets

To examine the efficacy of using long read sequence data for investigating ARG sharing dynamics, we compared the assembly metrics of contigs assembled using short or long sequence reads. The long sequence reads of the matched five *E. coli* isolates were generated using Oxford Nanopore Technology MinION Platform and provided by Broad, along with the paired-end Illumina reads. We generated the simulated PIC-seq *E. coli* dataset for long reads using the methods described for the short reads.

In addition to the contigs assembled using only short reads, we used two additional approaches: (1) assembling using short reads first, then correcting with long reads and (2) first assembling long reads and then polishing the resulting contigs with the short reads mapped onto them. Binning of sequence reads to the reference genomes identified by StrainGE was performed before assembly, as described for the short reads. To assemble contigs starting with short reads and then correct the resulting contigs with long reads, we used HybridSPAdes v3.15.4 (ref. ^65^) with default parameters. For the assembly of contigs beginning with long reads, we used metaFlye v.2.8.1 (ref. ^66^) with default parameters, followed by medaka v1.7.0 for error correction in the contigs, using the same long reads as input. Short reads were then mapped on to the contigs using bbmap^67^, and the resulting alignment files were used to polish errors in the contigs using pilon v1.24 (ref. ^68^). The assembly metrics of the contigs were estimated by using MetaQUAST v5.2.0 (ref. ^69^), based on reference genomes provided as the complete genomes of *E. coli* isolates used for simulated datasets.

### Statistical analysis

All statistical analyses in this study were carried out using R version 4.2.1 (ref. ^70^). All collected samples were included in the analyses, with no exclusions. The normality of the data distribution was tested before applying appropriate statistical tests. The differences in mean strain-sharing rates were compared using a two-sided nonparametric permutation test, with 1,000 iterations of resampling. Resistome similarity was estimated by Jaccard similarity of ARG cluster compositions, defined as the number of unique ARG clusters shared divided by the total number of unique ARG clusters from both samples. The pairwise Mann–Whitney *U* test was used for comparing resistome similarities between sample types, using the ‘wilcox.test’ command from the ‘stats’ package in R^70^. All *P* values were adjusted accordingly using Benjamini–Hochberg correction. Finally, the correlation analyses among strain-sharing and resistome similarity patterns were performed using Spearman’s rank correlation method, as implemented in the ‘cor.test’ function from the ‘stats‘ package in R^70^.

### Reporting summary

Further information on research design is available in the Nature Portfolio Reporting Summary linked to this article.

## Supplementary information

Supplementary Information Supplementary Figs. 1–4, Tables 1–5 and 7–9 and Notes.

Reporting Summary

Supplementary Table 6 Relative frequency of ARG clusters with predicted mobility.

Source Data Supplementary Fig. 1 Source data for Supplementary Fig. 1.

Source Data Supplementary Fig. 2 Source data for Supplementary Fig. 2.

Source Data Supplementary Fig. 3 Source data for Supplementary Fig. 3.

## Source data

Source Data Fig. 1 Source data for Fig. 1.

Source Data Fig. 2 Source data for Fig. 2.

Source Data Fig. 3 Source data for Fig. 3.

Source Data Fig. 4 Source data for Fig. 4.

Source Data Fig. 5 Source data for Fig. 5.

Source Data Fig. 6 Source data for Fig. 6.

Source Data Extended Data Fig. 2 Source data for Extended Data Fig. 2.

Source Data Extended Data Fig. 3 Source data for Extended Data Fig. 3.

Source Data Extended Data Fig. 4 Source data for Extended Data Fig. 4.

Source Data Extended Data Fig. 6 Source data for Extended Data Fig. 6.

Source Data Extended Data Fig. 7 Source data for Extended Data Fig. 7.

Source Data Extended Data Fig. 8 Source data for Extended Data Fig. 8.

Source Data Extended Data Fig. 9 Source data for Extended Data Fig. 9.

## Figures and Tables

**Extended Data Table 1:**
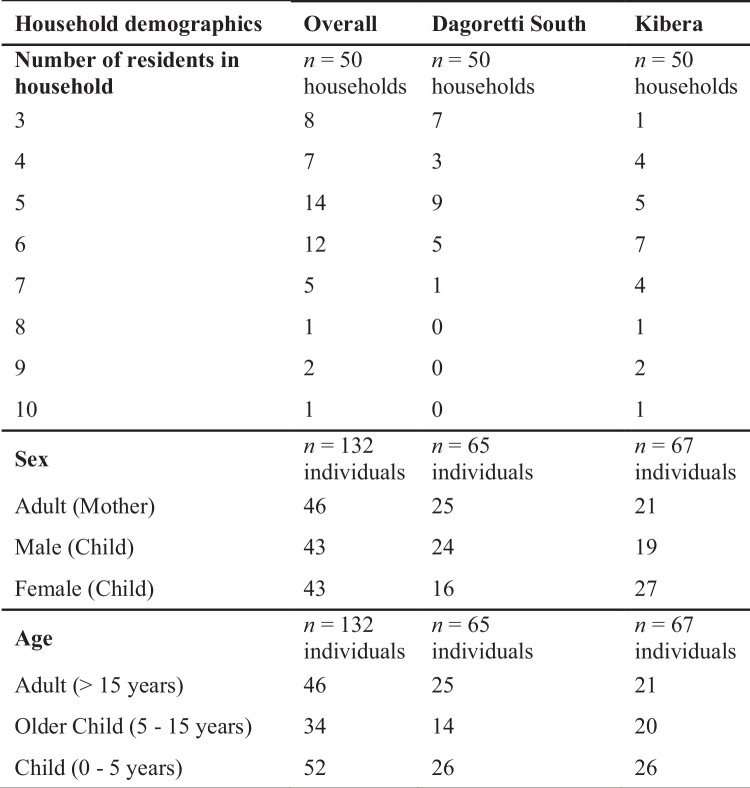
Demographics of enrolled households (n = 50) in Dagoretti South and Kibera

## Data Availability

All raw sequence data were deposited in the NCBI database under BioProject accession number PRJNA1126668. Publicly available reference databases used in this study include the NCBI RefSeq database (www.ncbi.nlm.nih.gov/refseq), the Virulence Factor Database (www.mgc.ac.cn/VFs/), mobileOG-db release beatrix-1.6 (https://mobileogdb.flsi.cloud.vt.edu), and the CARD v3.2.4 (https://card.mcmaster.ca). Source data are provided with this paper.

## Code availability

Custom R scripts used for the analysis are available at https://github.com/danielkim617/E_coli_strain_tracking.
