# Supplementary material for: Contaminated drinking water facilitates Escherichia coli strain-sharing within households in urban informal settlements
Source: Nat Microbiol. 2025 May 1;10(5):1198–209. doi: 10.1038/s41564-025-01986-w (PMC12055605; doi:10.1038/s41564-025-01986-w)
Supplement: Supplementary file 1 — Supplementary Figs. 1–4, Tables 1–5 and 7–9 and Notes. [file 41564_2025_1986_MOESM1_ESM.pdf]

nature microbiology

Supplementary information

https://doi.org/10.1038/s41564-025-01986-w

Contaminated drinking water facilitates *Escherichia coli* strain-sharing within households in urban informal settlements

In the format provided by the authors and unedited

Table of Contents:

Supplementary Notes

Supplementary Figures (Figures S1–S4)

Supplementary Tables (Tables S1–S9)

Supplementary notes

Household characteristics

Households in Dagoretti South were more likely to have latrines inside their compound (76%; 19/25 households) compared to Kibera (20%; 5/25 households), where the majority relied on public facilities located >5 meters away outside of the household compound (64%; 16/24 households) (Supplementary Table 8). Study staff observed less animal feces near household soil sampling areas in Dagoretti South (24%; 6/25 households) than in Kibera (48%; 12/25 households).

Strain identification of simulated PIC-seq datasets

The characteristics of *E. coli* strains used for generating the simulated PIC-seq data were analysed based on their complete genome sequences. Genome sizes across the strains ranged from 4.7 to 5.3 Mbp. The isolate GTEN 247 had the highest number of plasmids while no plasmid sequences were detected in the GTEN 378 isolate (Supplementary Table 9). In terms of phylogroup classification, the *E. coli* strains consisted of phylogroups commonly observed in real samples, with the isolates GTEN 291 and GTEN 293 assigned to identical phylogroup A (Supplementary Table 9). The range of unique ARG clusters identified across the strains was comparable, varying from 45 to 57. The GTEN 378 isolate had the lowest number ARGs predicted to be mobile, possibly due to the absence of plasmid. When individual strain identification was conducted on the Illumina sequences for each isolate, distinct strains were identified for each one.

Subsequent strain identification was conducted on simulated PIC-seq datasets generated by pooling sequence reads from *E. coli* strains in varying proportions. In all datasets, only four strains were identifiable, and the reference strain initially associated with GTEN 291, which was *E. coli* NCTC9087, was not identified, possibly due to high similarities

(> 99.0% ANI) between GTEN 291 and GTEN 293 (Supplementary Table 7). Except for GTEN 291, the trends of the relative abundance of the identified strains and the pooled ratio were generally in good agreement.

Benchmarking of reconstructed resistomes

The purpose of performing PIC-seq on *E. coli* isolates was not only to have a higher number of strains investigated for strain-sharing events but also to examine the resistomes with their genomic contexts. Sequence data comprising up to five different isolates is expected to yield better contig reconstruction compared to sequence data from more complex microbial communities. However, the assembly process could be challenging in deconvoluting pooled strains as the strains were highly similar. We employed the assembly approach suggested in plate sweep metagenomics, which involves a preliminary binning process based on reference strains before *de novo* assembly. In addition to assembly of binned reads, an additional *de novo* assembly was performed on the reads that did not map to any of the reference genomes, and ARGs and MGEs were annotated on the entire contigs. We performed benchmarking tests on this bioinformatics pipeline to evaluate its performance in reconstructing the resistome of samples, using simulated datasets. We also compared the resistomes reconstructed from contigs that were *de novo* assembled without a binning process to evaluate whether the binning process enhanced the accuracy of resistome reconstruction.

The assembly incorporating a binning process consistently outperformed those that did not in reconstructing resistomes across all simulated datasets. With a clustering at 100% nucleotide identity, the simulated dataset was expected to contain 212 overall ARG clusters, comprising 118 non-mobile and 94 mobile ARG clusters (Supplementary Fig. 2). In resistomes with a binning process, the number of overall true-positive ARG clusters, which aligned with those from reference genomes, ranged from 173 to 189 (accounting for 81.6% to 89.2%). In contrast, resistomes without a binning process only had 32 to 59 overall true-positive ARG clusters (accounting for 15.1% to 27.8%) (Supplementary Fig. 2). The number of false-positive overall ARG clusters, defined as those deviating from ARG sequences in the reference genomes, was comparable between resistomes reconstructed with and without a binning approach in the simulated datasets of ratio 1, 2, and 3. However, in the simulated datasets with ratios 4, 5, and 6, which had at least a 100-fold difference in the pooling ratios between strains, the number of false-positive ARG clusters was significantly lower in resistomes reconstructed without the binning process (fewer than 10 instances).

The same trends were observed in both non-mobile and mobile resistomes. The non-mobile resistome had a higher number of true-positive ARG clusters, while the mobile resistome had a lower number of false-positive ARG clusters.

To determine which approach more accurately captures the true resistome, we calculated the Jaccard similarity between the resistomes derived from the simulated datasets and the reference genomes. In all the simulated datasets, resistomes reconstructed using the binning process exhibited at least a 1.9-fold higher Jaccard similarity to the reference compared to those without binning. This suggests that the incorporation of a binning process in the assembly pipeline significantly enhances the accuracy of resistome reconstruction in this study. (Supplementary Fig. 3).

Zoonotic strain-sharing

It has been hypothesized that domestic animal exposure increases the risk of zoonosis.[1,2] While we rarely observed direct zoonotic sharing events in our study population, we documented that poultry-soil strain-sharing was more frequent *within* households compared to *between* households. In our urban study context, rare poultry-human strain-sharing within households could be explained by infrequent exchange of strains between humans and soil (Fig 3). Stored water would have likely played a minimal role in poultry-human strain- sharing, as animals were given water different from humans, as evidenced by the absence of strain-sharing between animals and stored water. Previous research in rural and/or urban regions of Kenya,[3,4] Bangladesh,[1,5] and India[6] has indicated that exposure to domesticated animals increases environmental fecal bacteria contamination, evidenced by the higher prevalence of animal versus human fecal markers in soil and drinking water. Given that environment-associated strain-sharing rates with humans or animals were significantly higher *within* households versus *between* households, our findings, highlighting the critical role of the environment in bacterial transmission. A few human-animal strain-sharing events were observed *between* households at a rate not significantly different from that *within* households, consistent with a previous study in Kenya, which found that sharing between humans and livestock was not confined to the same households.[7] One potential route for strain-sharing across households could be the consumption of *E. coli*-contaminated chicken meat supplied locally, which has been reported to be prevalent in low-income settings in Nairobi.[8] Taken together with previous evidence, our results suggest that human-animal strain-sharing can occur at the community scale.

Profiling mobility of ARGs

Prevalence of plasmid-mediated ARGs like *bla*$_{TEM-1}$[9] and *aph(6)-Id*[10] indicates certain ARGs and MGEs are more prone to dissemination.[11] Our profiling of ARG mobility was not always possible due to insufficient contiguity of contigs assembled from short reads. While long-read Oxford Nanopore sequencing technology has the potential to identify the genomic context of ARGs,[12] our benchmarking showed that contigs assembled with short reads outperformed those with long reads in sequence accuracy (Supplementary Fig. 1). Hybrid assembly, leveraging both short and long reads, has been suggested as an alternative approach with better assembly quality,[13] however, the improvement in sequence contiguity and accuracy for hybrid assembly was marginal in our benchmarking.

Supplementary figures

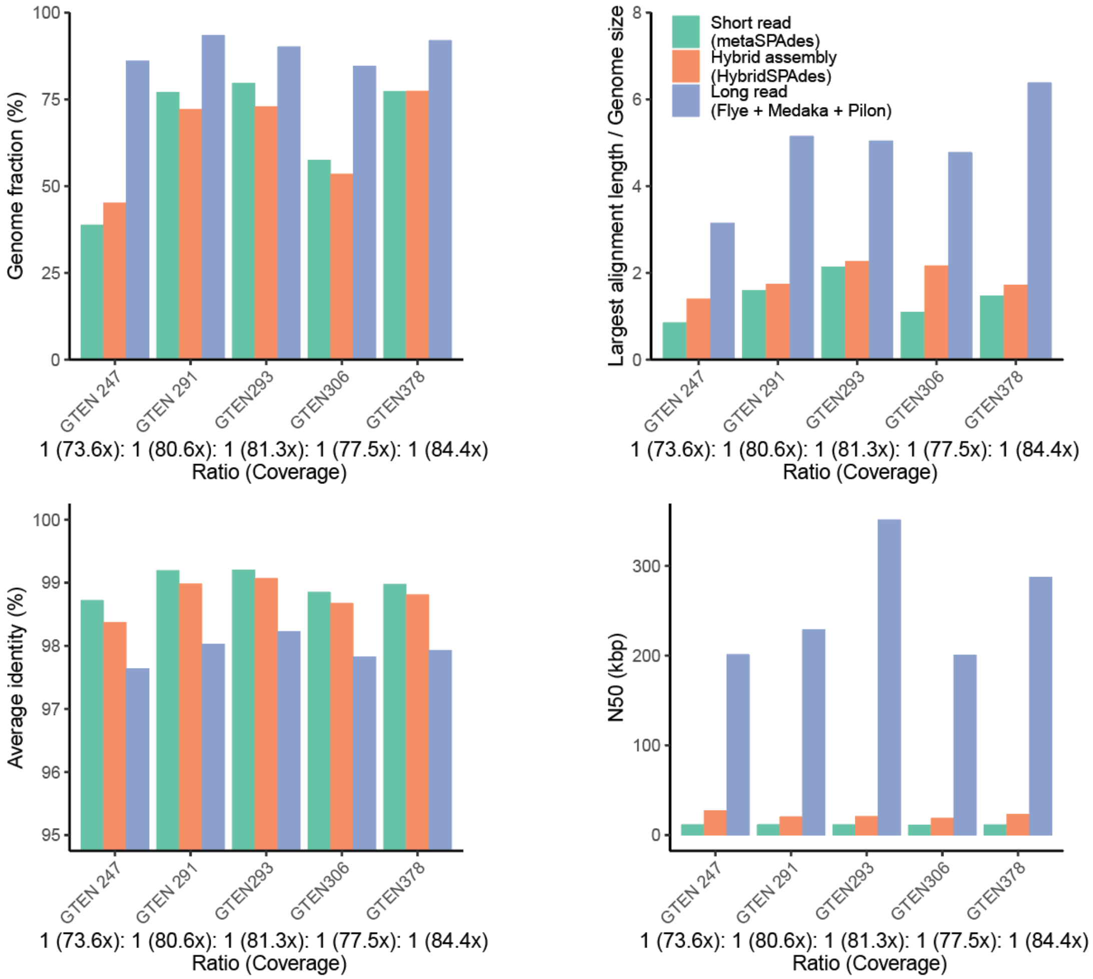

Supplementary Fig. 1 Assembly metrics for contigs assembled using short or long sequence reads. The simulated PIC-seq dataset of *E. coli* strains with an equal ratio of reads was used. The numbers in parentheses on x-axis specify the actual sequence coverage of each genome. In the fill color legend: *Short read* indicates the contigs assembled using only short reads; *Hybrid assembly* indicates the contigs initially assembled with short reads, subsequently error-corrected using long reads; *Long read* indicates the contigs that were assembled first using long read, then error-corrected with short reads aligned onto them.

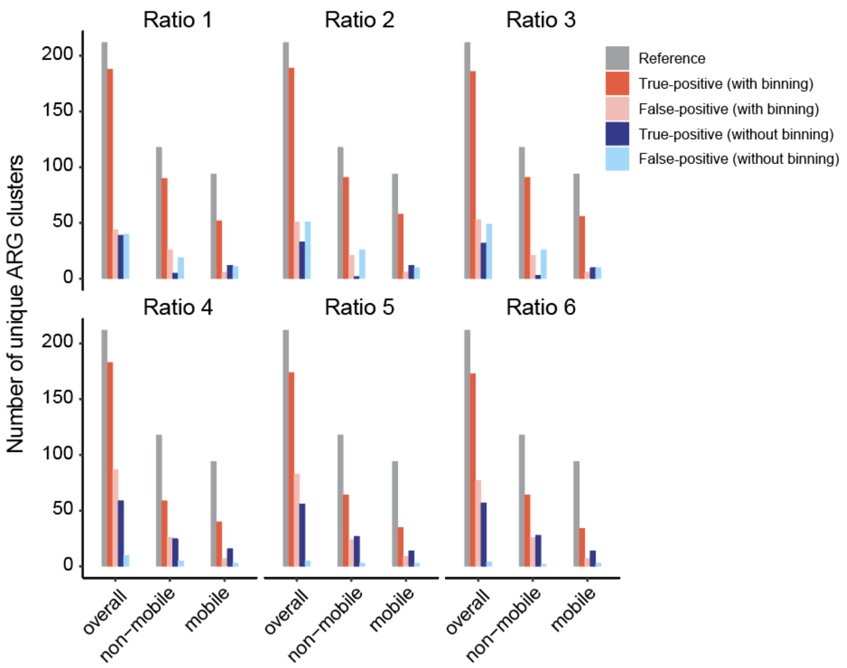

Supplementary Fig. 2 Number of unique ARG clusters in the benchmarking test of the bioinformatics pipelines using the simulated datasets.

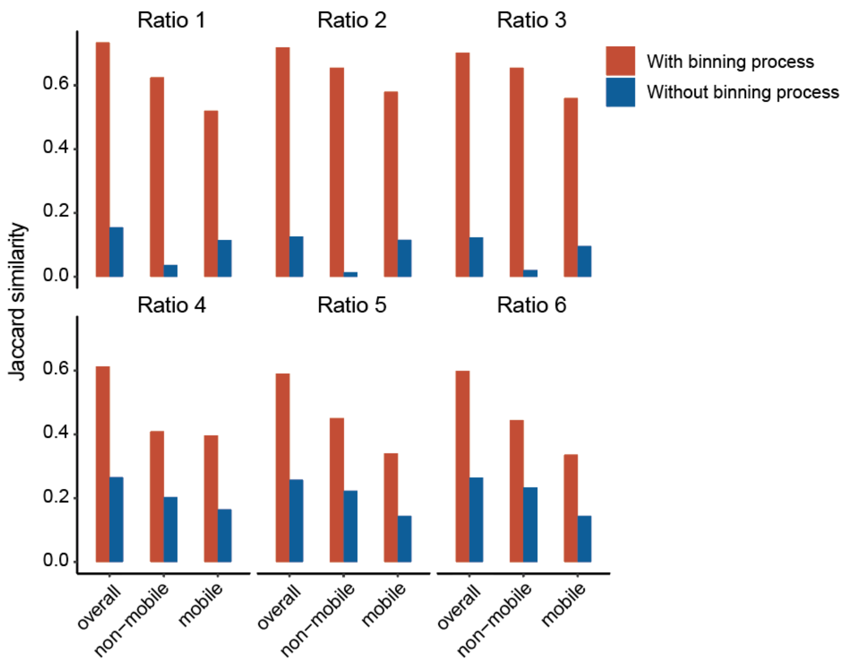

Supplementary Fig. 3 Jaccard similarity of resistomes based on ARG clusters in benchmarking compared to the reference.

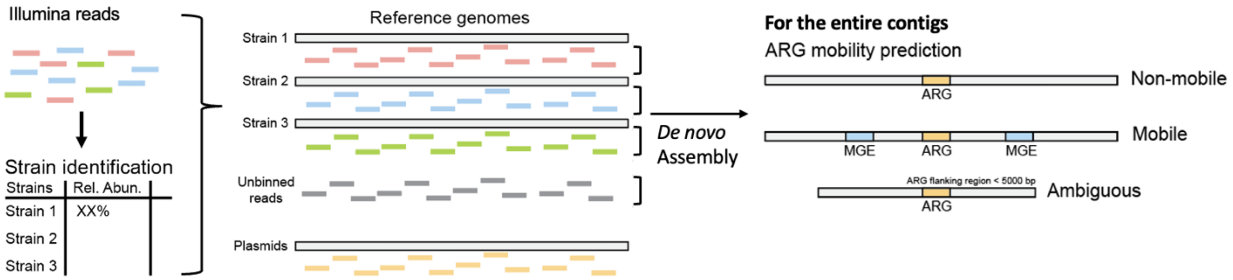

Supplementary Fig. 4 Schematic of the integrated bioinformatics pipeline that combines *de novo* assembly with reference-based binning for ARG annotation and mobility prediction.

Supplementary Tables

Supplementary Table 1 Household assets survey data for enrolled households (*n* = 50) in Dagoretti South and Kibera

Household assets	Overall (*n* = 50)	Dagoretti South (*n* = 25)	Kibera (*n* = 25)
Electricity	50 (100%)	25 (100%)	25 (100%)
Radio	40 (80%)	23 (92%)	17 (68%)
TV	47 (94%)	25 (100%)	22 (88%)
Mobile	49 (98%)	25 (100%)	24 (96%)
Clock	14 (28%)	5 (20%)	9 (36%)
Bicycle	9 (16%)	7 (28%)	2 (8%)
Motorcycle	5 (10%)	4 (16%)	1 (4%)
Stove	34 (68%)	16 (64%)	18 (72%)
Cooker	40 (80%)	22 (88%)	18 (72%)
Car	1 (2%)	1 (4%)	0 (0%)

Supplementary Table 2 Household animal practice survey data for enrolled households (n = 50) in Dagoretti South and Kibera

Household animal practice	Overall (n = 50)	Dagoretti South (n = 25)	Kibera (n = 25)
Purpose for poultry ownership			
Meat	45 (90%)	22 (88%)	23 (92%)
Eggs	39 (78%)	22 (88%)	17 (68%)
Income generation	31 (62%)	11 (44%)	20 (80%)
Selling bird	29 (58%)	10 (40%)	19 (76%)
Selling meat	2 (4%)	0 (0%)	2 (8%)
Selling eggs	7 (14%)	6 (24%)	1 (4%)
Pet	1 (2%)	0 (0%)	1 (4%)
For gift to a visitor	1 (2%)	0 (0%)	1 (4%)
Antibiotic use for poultry			
Yes	4 (8%)	2 (8%)	2 (8%)
Poultry and canine entering house			
Always / Often	13 (26%)	3 (12%)	10 (40%)
Sometimes	15 (30%)	10 (40%)	5 (20%)
Never	22 (44%)	12 (48%)	10 (40%)
Feces near household soil sampling area			
Yes	18 (36%)	6 (24%)	12 (48%)

Supplementary Table 3 Household drinking water survey data for enrolled households (n = 50) in Dagoretti South and Kibera

Household drinking water	Overall	Dagoretti South	Kibera
***E. coli* contamination in source water**	n = 37	n = 16	n = 21
Yes	2	2	0
No	26	9	17
Source water not collected (unavailable supply)	4	0	4
Source water not collected (distant location)	5	5	0
**Drinking water source types**	n = 50	n = 25	n = 25
Borehole	11 (22%)	11 (44%)	0 (0%)
Piped water into yard/plot	5 (10%)	0 (0%)	5 (20%)
Piped water outside the compound	31 (62%)	11 (44%)	20 (80%)
Tanker truck	3 (6%)	3 (12%)	0 (0%)
**Post water management**	n = 50	n = 25	n = 25
Bottled chlorine	3 (6%)	1 (4%)	2 (8%)
Boiling	5 (10%)	1 (4%)	4 (16%)
Filter (ceramic, sand, composite)	1 (2%)	1 (4%)	0 (0%)
**Stored water container**	n = 50	n = 25	n = 25
Jerry can	25 (50%)	15 (60%)	10 (40%)
Plastic bucket	15 (30%)	8 (32%)	7 (28%)
Clear plastic bottled water	5 (10%)	1 (4%)	4 (16%)
Clear plastic container (e.g. juice container)	3 (6%)	1 (4%)	2 (8%)
Clay pot	1 (2%)	0 (0%)	1 (4%)
Roof tank/cistern	1 (2%)	0 (0%)	1 (4%)
**Whether the container was covered**	n = 50	n = 25	n = 25
Fully covered	41 (82%)	22 (88%)	19 (76%)
Not covered (large-mouthed container)	1 (2%)	0 (0%)	1 (4%)
Not covered (narrow-mouthed container)	7 (14%)	3 (12%)	4 (16%)
Other	1 (2%)	0 (0%)	1 (4%)
**How to extract drinking water from the stored water container**	n = 50	n = 25	n = 25
Water poured from container	29 (58%)	16 (64%)	13 (52%)
Container/glass dipped into water container	15 (30%)	7 (28%)	8 (32%)
Water poured from tap/handpump	5 (10%)	2 (8%)	3 (12%)
Ladle used to obtain water	1 (2%)	0 (0%)	1 (4%)

Supplementary Table 4 Pairwise permutation test of mean strain-sharing rates among sample types within and between households.

Comparison	Sharing type 1	Sharing type 2	Mean rate (Sharing type1)	Mean rate (Sharing type2)	*p*	adj. *p*
Within	Human-Animal	Animal-Household soil	0.00166	0.02941	0.056	0.104
	Human-Animal	Animal-Drinking water	0.00166	0.00000	0.904	0.904
	Human-Animal	Human-Household soil	0.00166	0.00000	0.835	0.904
	Human-Animal	Human-Drinking water	0.00166	0.06463	0.003	0.027
	Animal-Household soil	Animal-Drinking water	0.02941	0.00000	0.042	0.104
	Animal-Household soil	Human-Household soil	0.02941	0.00000	0.180	0.270
	Animal-Household soil	Human-Drinking water	0.02941	0.06463	0.283	0.364
	Animal-Drinking water	Human-Household soil	0.00000	0.00000		-
	Animal-Drinking water	Human-Drinking water	0.00000	0.06463	0.009	0.041
	Human-Household soil	Human-Drinking water	0.00000	0.06463	0.056	0.104
Between	Human-Animal	Animal-Household soil	0.00128	0.00214	0.403	0.734
	Human-Animal	Animal-Drinking water	0.00128	0.00089	0.625	0.781
	Human-Animal	Human-Household soil	0.00128	0.00123	0.944	0.944
	Human-Animal	Human-Drinking water	0.00128	0.00030	0.117	0.390
	Animal-Household soil	Animal-Drinking water	0.00214	0.00089	0.242	0.605
	Animal-Household soil	Human-Household soil	0.00214	0.00123	0.476	0.734
	Animal-Household soil	Human-Drinking water	0.00214	0.00030	0.105	0.390
	Animal-Drinking water	Human-Household soil	0.00089	0.00123	0.770	0.856
	Animal-Drinking water	Human-Drinking water	0.00089	0.00030	0.514	0.734
	Human-Household soil	Human-Drinking water	0.00123	0.00030	0.095	0.390

Supplementary Table 5 Comparison of strain-sharing rates within households with and without *E. coli* contamination in stored drinking water. *P*-values were calculated using a two-sided permutation test and were not adjusted, as each comparison was analyzed separately.

Sharing type (within households)	Mean strain-sharing rate		*p*
	E. coli positive in stored water	*E. coli* negative in stored water	
Human-Human	0.317	0.0897	0.020
Poultry-Poultry	0.167	0.150	0.992
Human-Poultry	0.000	0.000	-
Human-Canine	0.000	0.000	-
Human-Drinking water	0.144	0.000	0.004
Human-Household soil	0.000	0.000	-
Poultry-Canine	0.000	0.077	1.000
Poultry-Drinking water	0.000	0.000	-
Poultry-Household soil	0.075	0.000	0.249
Canine-Drinking water	0.000	0.000	-
Canine-Household soil	0.000	0.000	-
Drinking water- Household soil	0.100	0.000	0.476

Supplementary Table 6 Relative frequency of ARG clusters with predicted mobility (in a separate Excel sheet)

Supplementary Table 7 Description of the simulated datasets for benchmarking

		GTEN 247	GTEN 291	GTEN 293	GTEN 306	GTEN 378
	Genome size (Mbp)	5.5	5.0	4.9	5.2	4.7
	Expected coverage	73.6X	80.6X	81.3X	77.5X	84.4X
	StrainGST identification	*E. coli* TUM20902	-	*E. coli* MB19	*E. coli* 14EC033	*E. coli* 2011C-3911
	StrainGST relative abundance (%)	15.5	-	24.6	28.3	24.9
Ratio2	Pooled ratio	1	0.1	1	1	1
	Expected coverage	88.5X	9.8X	99.2X	95.4X	103.0X
	StrainGST identification	*E. coli* TUM20902	-	*E. coli* MB19	*E. coli* 14EC033	*E. coli* 2011C-3911
	StrainGST relative abundance (%)	17.3	-	19.3	30.7	25.6
Ratio3	Pooled ratio	1	0.025	1	1	1
	Expected coverage	90.2X	2.5X	101.0X	96.3X	104.9X
	StrainGST identification	*E. coli* TUM20902	-	*E. coli* MB19	*E. coli* 14EC033	*E. coli* 2011C-3911
	StrainGST relative abundance (%)	17.5	-	18.9	30.9	25.6

Ratio4	Pooled ratio	0.1	1	1	1	10
	Expected coverage	2.8X	30.8X	31.0X	29.6X	322.3X
	StrainGST identification	*E. coli* TUM20902	-	*E. coli* MB19	*E. coli* 14EC033	*E. coli* 2011C-3911
	StrainGST relative abundance (%)	1.9	-	17.2	12.2	61.9
Ratio5	Pooled ratio	0.1	0.1	1	1	10
	Expected coverage	3.0X	3.3X	33.3X	31.8X	346.1X
	StrainGST identification	*E. coli* TUM20902	-	*E. coli* MB19	*E. coli* 14EC033	*E. coli* 2011C-3911
	StrainGST relative abundance (%)	1.7	-	13.1	12.2	66.1
Ratio6	Pooled ratio	0.1	0.025	1	1	10
	Expected coverage	3.0X	0.8X	33.5X	32.0X	348.2X
	StrainGST identification	*E. coli* TUM20902	-	*E. coli* MB19	*E. coli* 14EC033	*E. coli* 2011C-3911
	StrainGST relative abundance (%)	1.6	-	12.8	12.1	66.7

Supplementary Table 8 Household latrine survey data for enrolled households (n = 50) in Dagoretti South and Kibera

Household latrine	Overall (n = 50)	Dagoretti South (n = 25)	Kibera (n = 25)
Location			
Inside compound	24 (48%)	19 (76%)	5 (2%)
Immediately outside compound (< 5 m away)	7 (14%)	4 (16%)	3 (12%)
Outside compound (> 5 m away)	18 (36%)	2 (8%)	16 (64%)
No answer	1 (2%)	0 (0%)	1 (4%)

Supplementary Table 9 Characteristics of the isolates used for the simulated datasets

Identifier	Genome size (Mbp)	Phylogroup	StrainGST identification	Number of plasmids	Number of unique ARG clusters		
					Overall	Non-mobile	Mobile
GTEN 247	5.5	D	*E. coli* TUM20902	4	57	30	27
GTEN 291	5.0	A	*E. coli* NCTC9087	1	46	25	21
GTEN 293	4.9	A	*E. coli* MB19	2	44	27	17
GTEN 306	5.2	E	*E. coli* 14EC033	3	47	19	28
GTEN 378	4.7	B1	*E. coli* 2011C-3911	0	45	30	15

Reference

1. Ercumen, A. et al. Animal feces contribute to domestic fecal contamination: evidence from *E. coli* measured in water, hands, food, flies, and soil in Bangladesh. *Environ. Sci. Technol.* 51, 8725–8734 (2017).

2. Zambrano, L. D., Levy, K., Menezes, N. P. & Freeman, M. C. Human diarrhea infections associated with domestic animal husbandry: a systematic review and meta-analysis. *Trans. R. Soc. Trop. Med. Hyg.* 108, 313–325 (2014).

3. Mosites, E. et al. Microbiome sharing between children, livestock and household surfaces in western Kenya. *PLoS One* 12, e0171017 (2017).

4. Swarthout, J. M. et al. Addressing Fecal Contamination in Rural Kenyan Households: The Roles of Environmental Interventions and Animal Ownership. *Environ. Sci. Technol.* (2024).

5. Harris, A. R. et al. Ruminants contribute fecal contamination to the urban household environment in Dhaka, Bangladesh. *Environ. Sci. Technol.* 50, 4642–4649 (2016).

6. Schriewer, A. et al. Human and animal fecal contamination of community water sources, stored drinking water and hands in rural India measured with validated microbial source tracking assays. *Am. J. Trop. Med. Hyg.* 93, 509 (2015).

7. Hassell, J. M., Begon, M., Ward, M. J. & Fèvre, E. M. Urbanization and disease emergence: dynamics at the wildlife–livestock–human interface. *Trends Ecol. Evol.* 32, 55–67 (2017).

8. Odwar, J. A., Kikuvi, G., Kariuki, J. N. & Kariuki, S. A cross-sectional study on the microbiological quality and safety of raw chicken meats sold in Nairobi, Kenya. *BMC Res. Notes* 7, 1–8 (2014).

9. Brown, N. G., Shanker, S., Prasad, B. V. & Palzkill, T. Structural and biochemical evidence that a TEM-1 β-lactamase N170G active site mutant acts via substrate-assisted catalysis. *J. Biol. Chem.* 284, 33703–33712 (2009).

10. Khezri, A., Avershina, E. & Ahmad, R. Plasmid identification and plasmid-mediated antimicrobial gene detection in Norwegian isolates. *Microorganisms* 9, 52 (2020).

11. Ellabaan, M. M., Munck, C., Porse, A., Imamovic, L. & Sommer, M. O. Forecasting the dissemination of antibiotic resistance genes across bacterial genomes. *Nat. Commun.* 12, 2435 (2021).

12. Peter, S. et al. Tracking of antibiotic resistance transfer and rapid plasmid evolution in a hospital setting by Nanopore sequencing. *mSphere* 5, e00525-20 (2020).

13. Brown, C. L. et al. Critical evaluation of short, long, and hybrid assembly for contextual analysis of antibiotic resistance genes in complex environmental metagenomes. *Sci. Rep.* 11, 3753 (2021).
