# Supplementary material for: Contaminated drinking water facilitates Escherichia coli strain-sharing within households in urban informal settlements
Source: Nat Microbiol. 2025 May 1;10(5):1198–209. doi: 10.1038/s41564-025-01986-w (PMC12055605; doi:10.1038/s41564-025-01986-w)
Supplement: Supplementary file 2 — Reporting Summary [file 41564_2025_1986_MOESM2_ESM.pdf]

nature portfolio

Corresponding author(s): Amy J. Pickering

Last updated by author(s): Mar 4, 2025

Reporting Summary

Nature Portfolio wishes to improve the reproducibility of the work that we publish. This form provides structure for consistency and transparency in reporting. For further information on Nature Portfolio policies, see our Editorial Policies and the Editorial Policy Checklist.

Statistics

For all statistical analyses, confirm that the following items are present in the figure legend, table legend, main text, or Methods section.

n/a	Confirmed	
☐	☒	The exact sample size (*n*) for each experimental group/condition, given as a discrete number and unit of measurement
☐	☒	A statement on whether measurements were taken from distinct samples or whether the same sample was measured repeatedly
☐	☒	The statistical test(s) used AND whether they are one- or two-sided<br>*Only common tests should be described solely by name; describe more complex techniques in the Methods section.*
☐	☒	A description of all covariates tested
☐	☒	A description of any assumptions or corrections, such as tests of normality and adjustment for multiple comparisons
☐	☒	A full description of the statistical parameters including central tendency (e.g. means) or other basic estimates (e.g. regression coefficient) AND variation (e.g. standard deviation) or associated estimates of uncertainty (e.g. confidence intervals)
☐	☒	For null hypothesis testing, the test statistic (e.g. *F*, *t*, *r*) with confidence intervals, effect sizes, degrees of freedom and *P* value noted<br>*Give P values as exact values whenever suitable.*
☒	☐	For Bayesian analysis, information on the choice of priors and Markov chain Monte Carlo settings
☒	☐	For hierarchical and complex designs, identification of the appropriate level for tests and full reporting of outcomes
☐	☒	Estimates of effect sizes (e.g. Cohen's *d*, Pearson's *r*), indicating how they were calculated

Our web collection on statistics for biologists contains articles on many of the points above.

Software and code

Policy information about availability of computer code

Data collection	All survey data were collected on electronic tablets by SurveyCTO (Dobility, Cambridge, MA, USA).
Data analysis	We used the following open-source software for DNA sequence analysis (all available through GitHub): FastQC v0.12.1, Trimmomatic v0.39, StrainGE v1.3.3, bwa mem v0.7.17, Prokka v1.14.5, Roary v3.13.0, MAFFT, SNP-sites v2.5.1, RAxML v8.2.12, ClermonTyping v20.03, mlst v2.22.1, ABRicate v1.0.1, metaSPAdes v3.15.4, mSWEEP v2.0.0, mGEMs v1.3.0, Prodigal v2.6.3, DIAMOND blastp v2.0.14.152, mobileOG-pl, MOB-suite v3.1.0, seqtk v1.3, HybridSPAdes v3.15.4, metaFlye v.2.8.1, medaka v1.7.0, bbmap, pilon v1.24, and MetaQUAST v5.2.0. The analyses were conducted in R v4.2.1 using codes provided in the GitHub repository (https://github.com/danielkim617/E_coli_strain_tracking)

For manuscripts utilizing custom algorithms or software that are central to the research but not yet described in published literature, software must be made available to editors and reviewers. We strongly encourage code deposition in a community repository (e.g. GitHub). See the Nature Portfolio guidelines for submitting code & software for further information.

Data

Policy information about availability of data

All manuscripts must include a data availability statement. This statement should provide the following information, where applicable:

- Accession codes, unique identifiers, or web links for publicly available datasets
- A description of any restrictions on data availability
- For clinical datasets or third party data, please ensure that the statement adheres to our policy

All raw sequence data were deposited in the NCBI database under BioProject accession number PRJNA1126668. Publicly available reference databases used in this study include the NCBI RefSeq database (www.ncbi.nlm.nih.gov/refseq), the Virulence Factor Database (VFDB) (www.mgc.ac.cn/VFs/), mobileOG-db release beatrix-1.6 (mobileogdb.flsi.cloud.vt.edu), and the Comprehensive Antibiotic Resistance Database (CARD) v3.2.4 (card.mcmaster.ca).

Research involving human participants, their data, or biological material

Policy information about studies with human participants or human data. See also policy information about sex, gender (identity/presentation), and sexual orientation and race, ethnicity and racism.

Reporting on sex and gender

We collected sex information of the participants with consent.

Reporting on race, ethnicity, or other socially relevant groupings

We report on household assets (e.g., availability of electricity, TV, mobile phone, stove), latrine access, domesticated animal practices, and water treatment status of the households based on the survey data to investigate if these factors affect the level of bacterial strain-sharing. We did not record ethnicity in the study.

Population characteristics

We collected samples from 50 poultry-owning households with at least one child under 5 years old in two subcounties in Nairobi, Dagoretti South (n = 25) and Kibera (n = 25). All households in Kibera used chlorinated piped water, while none of the water sources in Dagoretti South were chlorinated. Dagoretti South and Kibera had similar wealth indices, as measured by household assets like electricity, TV, mobile phones, and stoves. Poultry ownership in both areas was mainly for nutrient provision via meat and eggs or income generation. Over half of the respondents allowed poultry and canines into their homes, with study staff observing less animal feces near household soil sampling areas in Dagoretti South compared to Kibera.

Recruitment

Households were personally visited and randomly selected for the study based on specific criteria: poultry ownership and the presence of at least one child under the age of 5 years. If more than one household was eligible within a compound, one was randomly selected for participation. Eligible households were then invited to participate, with written informed consent obtained from each adult participant. For children, both child assent and parental written consent were obtained.

Ethics oversight

The study received ethical approval from the Kenya Medical Research Institute (KEMRI) Scientific and Ethics Review Unit (12/3823) and the Tufts Health Sciences Institutional Review Board (13205). Additionally, a research permit was granted by the Kenyan National Commission for Science, Technology, and Innovation.

Note that full information on the approval of the study protocol must also be provided in the manuscript.

Field-specific reporting

Please select the one below that is the best fit for your research. If you are not sure, read the appropriate sections before making your selection.

☒ Life sciences ☐ Behavioural & social sciences ☐ Ecological, evolutionary & environmental sciences

For a reference copy of the document with all sections, see nature.com/documents/nr-reporting-summary-flat.pdf

Life sciences study design

All studies must disclose on these points even when the disclosure is negative.

Sample size

No sample size calculation was conducted for this observational cross-sectional study. We used a permutation test, a conservative statistical method, to identify statistically significant findings, including an association between contaminated water and the transmission of bacterial strains and antibiotic-resistance genes.

Data exclusions

No data were excluded from the analyses.

Replication

Since our analysis involves processing sequencing data using computational code, the results remain consistent each time the code is executed. All data and code needed for replication are available.our analyses are publicly available without restrictions.

Randomization

Urban informal settlements in Kenya are typically organized into compounds where multiple households share a common courtyard. In this cross-sectional study, one household was randomly selected and enrolled from each compound that owned poultry. Since this study was not a randomized controlled trial, we did not allocate households into experimental or control groups. One study community had access to chlorinated water while one did not.

Blinding	No blinding was performed, as the study was designed as an observational cross-sectional study.

Reporting for specific materials, systems and methods

We require information from authors about some types of materials, experimental systems and methods used in many studies. Here, indicate whether each material, system or method listed is relevant to your study. If you are not sure if a list item applies to your research, read the appropriate section before selecting a response.

Materials & experimental systems

n/a	Involved in the study
☒	☐ Antibodies
☒	☐ Eukaryotic cell lines
☒	☐ Palaeontology and archaeology
☐	☒ Animals and other organisms
☒	☐ Clinical data
☒	☐ Dual use research of concern
☒	☐ Plants

Methods

n/a	Involved in the study
☒	☐ ChIP-seq
☒	☐ Flow cytometry
☒	☐ MRI-based neuroimaging

Animals and other research organisms

Policy information about studies involving animals; ARRIVE guidelines recommended for reporting animal research, and Sex and Gender in Research

Laboratory animals	The study did not involve laboratory animals.
Wild animals	The study did not involve wild animals.
Reporting on sex	Sex was not considered in the study design.
Field-collected samples	We collected poultry cloacal swabs and canine fecal samples from each household. A trained veterinary student administered the poultry cloacal swabs and placed them in storage tubes filled with Cary-Blair transport medium, which were kept in a cooler for transport to the lab. To collect canine feces, the top layer of fresh feces from the center of the pile was transferred into a 50 mL centrifuge tube with a sterile plastic scoop. The collected samples were then streaked onto Tryptone Bile X-glucuronide (TBX) agar plates to culture Escherichia coli colonies.
Ethics oversight	The study received ethical approval from the Kenya Medical Research Institute (KEMRI) Scientific and Ethics Review Unit (12/3823) and the Tufts Health Sciences Institutional Review Board (13205). Additionally, a research permit was granted by the Kenyan National Commission for Science, Technology, and Innovation.

Note that full information on the approval of the study protocol must also be provided in the manuscript.

Plants

Seed stocks	*Report on the source of all seed stocks or other plant material used. If applicable, state the seed stock centre and catalogue number. If plant specimens were collected from the field, describe the collection location, date and sampling procedures.*
Novel plant genotypes	*Describe the methods by which all novel plant genotypes were produced. This includes those generated by transgenic approaches, gene editing, chemical/radiation-based mutagenesis and hybridization. For transgenic lines, describe the transformation method, the number of independent lines analyzed and the generation upon which experiments were performed. For gene-edited lines, describe the editor used, the endogenous sequence targeted for editing, the targeting guide RNA sequence (if applicable) and how the editor was applied.*
Authentication	*Describe any authentication procedures for each seed stock used or novel genotype generated. Describe any experiments used to assess the effect of a mutation and, where applicable, how potential secondary effects (e.g. second site T-DNA insertions, mosiacism, off-target gene editing) were examined.*
